# Transient modes of zeolite surface growth from 3D gel-like islands to 2D single layers

**DOI:** 10.1038/s41467-018-04296-4

**Published:** 2018-05-29

**Authors:** Manjesh Kumar, Madhuresh K. Choudhary, Jeffrey D. Rimer

**Affiliations:** Department of Chemical and Biomolecular Engineering, University of Houston, Houston, TX 77204 USA

## Abstract

Zeolite crystallization occurs by multifaceted processes involving molecule attachment and nonclassical pathways governed by the addition of amorphous precursors. Here, we use scanning probe microscopy to monitor zeolite LTA crystallization in situ with a spatiotemporal resolution that captures dynamic processes in real time. We report a distinctive pathway involving the formation of gel-like islands from supersaturated solutions comprised of (alumino)silicate molecules. Three-dimensional assembly and evolution of these islands constitutes a unique mode of growth that differs from classical theories. Time-resolved imaging also reveals that growth can occur by (nearly) oriented attachment. At later stages of crystallization, a progressive transition to lower supersaturation shifts growth to a layered mechanism involving two-dimensional nucleation and spreading of layers. Here, we show that LTA crystallization occurs by multiple pathways, thereby reconciling putative hypotheses of growth mechanisms while also highlighting new modes of nonclassical crystallization that may prove relevant to other zeolites and related materials.

## Abstract (web summary)

While zeolites are industrially relevant as molecular sieves and catalysts, their growth mechanisms remain widely debated. Here, Rimer and colleagues probe the crystallization pathway of zeolite LTA with spatiotemporal resolution, identifying a distinctive nonclassical pathway, and demonstrating that growth is highly dependent on synthetic conditions.

## Introduction

Identifying pathway(s) of crystallization is critical to understanding, and ultimately controlling, the formation of natural^1^, synthetic^2^, or biological^3^ materials. It is increasingly evident that many crystalline materials exhibit nonclassical mechanisms involving the assembly and attachment of precursors that range in complexity from oligomers and liquid-like droplets to amorphous particles and small crystallites^4^. Examples include biominerals^5^, metals^6^, and metal oxides^7^, as well as microporous zeolites that grow from diverse precursors^8^ and by complex pathways involving monomer addition^9,10^, particle attachment^11–13^, and gel-to-crystal transformations^14,15^. Knowledge of zeolite crystallization is derived predominantly from ex situ studies that are insufficient to elucidate the mechanism(s) of growth. In this study, we focus on LTA (or zeolite A), which is a three-dimensional small-pore (0.42-nm) zeolite. Conventional synthesis of LTA results in a high-aluminum content (Si/Al = 1.0) that is ideal for adsorption and ion exchange^16^, whereas high-silica LTA (Si/Al > 10) has garnered interest as a catalyst owing to recent findings^17^ that Cu-exchanged LTA possesses unique properties for the selective catalytic reduction of NOx. Hypotheses of LTA crystallization derived from spectroscopy^18,19^, microscopy^20,21^, and molecular simulations^22,23^ postulate diverse pathways that include gel transformations^24^, precursor agglomeration and densification^25^, and the attachment of composite building units (CBUs)^26,27^. Such broad disparity of pathways underscores the need to reconcile the mode(s) of LTA crystallization over a range of synthesis conditions.

Herein, we use atomic force microscopy (AFM) to track the dynamics of LTA (100) surface growth. The growth media used for this study are the supernatants of a reported synthesis mixture^28^ after preheating for periodic times to generate a range of supersaturation with respect to silica. In situ AFM measurements at two different temperatures (35 and 45 °C) and variable supersaturation reveal multiple modes of growth that range from classical molecule-by-molecule addition to nonclassical pathways that, to our knowledge, have not been previously reported for zeolite crystallization. In this study, we show that time-resolved AFM imaging is able to capture the dynamics of these processes, thereby offering new insight into the mechanisms of LTA growth.

## Results

### Preparation of growth solutions with varying supersaturation

**Fig. 1:**
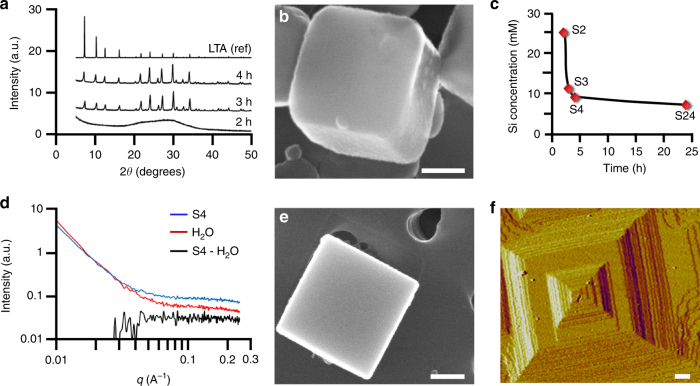
LTA crystals and growth solutions. **a** Powder X-ray diffraction patterns of solid precipitate obtained after heating growth solutions at 65 °C for various times. The LTA framework was confirmed using a reference (top pattern). **b** Electron micrograph of a LTA crystal after heating for 4 h at 65 °C. **c** Silicon concentration in solution obtained at various times (S2–S4 are used as growth solutions for in situ AFM). Additional elemental analysis is provided in Supplementary Table 1. **d** Small-angle X-ray scattering patterns of supernatant S4 and the background (water). Subtraction of the two patterns (black line) indicates the absence of nanoparticles (see Supplementary Fig. 2 for solutions S2 and S3). **e** Electron micrograph of a crystal prepared by heating supernatant S4 for 24 h at 45 °C. **f** AFM image of a LTA crystal surface (see also Supplementary Fig. 5) depicting a hillock with step heights of 1.2 nm, equivalent to the unit cell (*a* = *b* = *c* = 1.19 nm, *Pm-3m*). All scale bars are equal to 200 nm

LTA crystals are prepared with Na^+^ ions as inorganic structure-directing agents to facilitate the formation of a cubic lattice with tetrahedral sites that are occupied by Si and Al atoms supplied from colloidal silica and sodium aluminate, respectively. Growth solutions are comprised of amorphous precursors containing undissolved silica surrounded by a shell of alumina^29^. X-ray diffraction (Fig. 1a) reveals the onset of nucleation within 3 h of heating at 65 °C and the near completion of crystallization (Fig. 1b) after ~4 h. Extraction and analysis of the supernatant after periodic heating times confirms that supersaturated solutions (Fig. 1c and Supplementary Table 1) have sufficient nutrients for the nucleation and growth of cubic LTA crystals (Fig. 1e and Supplementary Fig. 1). Elemental analysis of the supernatant reveals a monotonic decrease in silicon concentration within the first 4 h of heating, followed by a gradual decrease to the equilibrium value after 24 h (solution S24 in Fig. 1c).

Solutions S2 to S4 in Fig. 1c are used as growth solutions for in situ measurements. A combination of small-angle X-ray scattering (Fig. 1d and Supplementary Fig. 2) and dynamic light scattering shows no trace of particulates in the supernatant, suggesting that these clear solutions (Supplementary Fig. 3) are predominantly comprised of soluble monomers and/or small oligomers. Prior studies have shown that (alumino)silicate particles or sol gels in LTA growth solutions are sites for heterogeneous nucleation^24^, while homogeneous nucleation has been observed in solutions devoid of amorphous precursors^30^. The clear solutions used in this study differ from those of other zeolites, such as silicalite-1, which are predominantly suspensions of nanoparticles (1–6 nm)^31–33^ that attach to crystal surfaces and undergo structural rearrangement to incorporate into the underlying crystal lattice^9^. As we report here, LTA crystallization occurs by an alternative mechanism.

### AFM measurements at low temperature

**Fig. 2:**
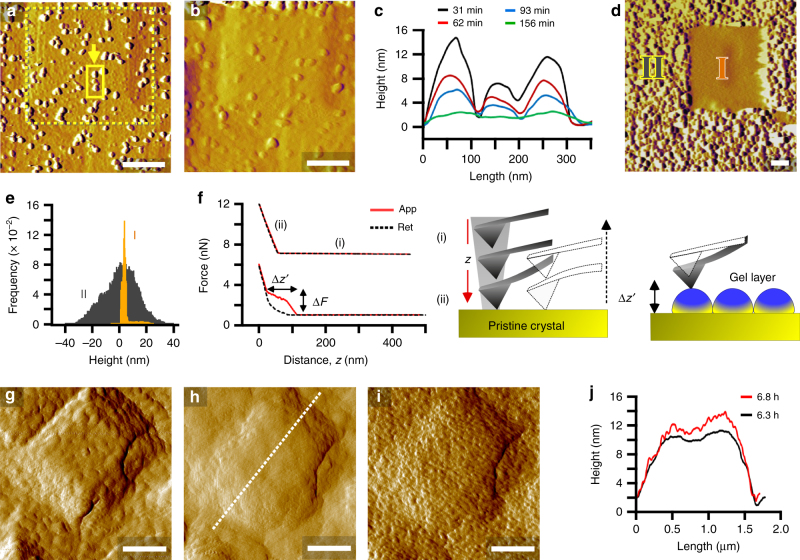
Three-dimensional growth of gel-like islands. **a**, **b** AFM amplitude mode images of a crystal surface at 35 °C in solution S2 (high supersaturation): **a** 30 min after reaching the set point temperature and **b** after 3 h of continuous imaging. **c** Time-resolved height profiles of the region highlighted by the arrow in **a**. **d** Enlarged scan area showing (I) a smooth region after 8 h of continuous imaging in tapping mode and (II) the surrounding rough region. **e** Statistical analysis of feature heights in regions I and II of image d (see also Supplementary Fig. 7). **f** CFM approach–retract curves as a function of distance *z* from substrates in solution S2 before heating (top) and after heating for 3 h (bottom). The schematics depict an AFM tip far from the surface (i) and in contact with the surface (ii). CFM on gel interfaces yields nonlinear approach curves with characteristic force Δ*F* and z-height Δ*z’*. **g–i** Amplitude mode images of a crystal surface in solution S3 at 35 °C; **g** 30 min after reaching the set point, **h** after 6.3 h of imaging, and **i** the same area after removing the AFM tip from the surface for 30 min and reimaging. **j** Height profiles along the dashed line in **h** for times 6.3 and 6.8 h (i.e., **h** and **i**, respectively). Scale bars are equal to 500 nm unless otherwise labeled

Crystal substrates prepared for AFM (Supplementary Fig. 4) are laden with hillocks (Fig. 1f and Supplementary Fig. 4) consisting of layers with step heights equal to the unit cell (Supplementary Fig. 5). The topography of LTA surfaces is similar to other zeolites extracted from their growth solutions at equilibrium (i.e., solubility). The appearance of unfinished layers suggests that growth occurs by layer advancement from the addition of monomers (or possibly small oligomers). Upon introduction of a highly supersaturated solution (S2) to an AFM liquid cell at room temperature, there is no apparent change in surface topography. Once the cell is heated at 35 °C, we observe the formation of islands (Fig. 2a) that render faceted hillocks rough and individual layers less defined with time. Continuous imaging in tapping mode leads to a smoothening of crystal surfaces (Fig. 2b and Supplementary Fig. 6) owing to the temporal reduction in island height (Fig. 2c). An enlarged image (Fig. 2d) clearly shows a smooth region (I in Fig. 2d) corresponding to an original scanning area and the surrounding rough region (II in Fig. 2d) outside of the previously imaged area. The rough region has a much broader distribution of surface feature heights than the former (Fig. 2e and Supplementary Fig. 7).

Elemental analysis (Supplementary Table 2) reveals that the amorphous islands contain more aluminum (Si/Al = 0.7). Ex situ studies of LTA crystal seeds grown in a S2 solution reveal a shift with increased heating time to the expected composition of LTA crystal surfaces (Si/Al = 1.1, Supplementary Table 2) along with the re-emergence of hillocks comprised of roughened steps (Supplementary Fig. 8). This suggests that growth occurs within the gel in close proximity to the crystal surface, thereby creating a growth front that advances normal to the gel–crystal interface. It is difficult to measure the rate of layer advancement by AFM owing to the absence of a constant baseline to accurately measure changes in height; however, bulk crystallization at 35 °C confirms that LTA nucleates and grows into fully crystalline cubes under conditions that mimic the AFM liquid cell (Supplementary Fig. 8).

A reduction of island height with AFM tip rastering implies the removal of loosely bound material from the crystal surface, which we attribute to a unique gel-like quality of the islands. Additional evidence is gleaned from chemical force microscopy (CFM) using a non-functionalized cantilever in S2 growth solution. CFM measurements prior to and after heating examine AFM tip interactions with pristine (crystalline) and gel layers, respectively (Supplementary Fig. 9). The approach curve for pristine surfaces is typical of most hard substrates, whereas the nonlinear profile on the gel layer (Fig. 2f) is similar to those produced by elastic substrates (e.g., lipids)^34,35^. Nonlinear approach curves (Supplementary Fig. 9e) have an average breakpoint force Δ*F* of 1.8 ± 0.6 nN and an approach distance Δ*z*’ of 52 ± 26 nm (Supplementary Fig. 10) that is commensurate with the height of surface features in AFM images (Fig. 2e). Moreover, analysis of the retraction curves reveals a higher tip–substrate adhesion force on rough surfaces (Supplementary Fig. 9f) and a hysteresis of approach–retraction curves, consistent with an elastic response of gel layers. To our knowledge, this is the first observation of nonclassical crystal growth involving molecularly dispersed solutes forming gel-like islands on crystal surfaces.

The presence of gel-like islands is prevalent on LTA surfaces at 35 °C. This is true even at reduced supersaturation (S3) where AFM tip-induced smoothening of crystal surfaces is observed (Fig. 2g, h; Supplementary Fig. 11 and Supplementary Movie 1). The latter is confirmed by removing the AFM tip from an area after continuous scanning and reengaging after a period of time (Fig. 2i) to show the recovery to a roughened crystal interface (Fig. 2j and Supplementary Fig. 12). One notable difference between gel-like islands formed from solutions of low (Fig. 2j) and high (Fig. 2e) supersaturation is larger feature sizes at the latter condition.

### AFM measurements at higher temperature

**Fig. 3:**
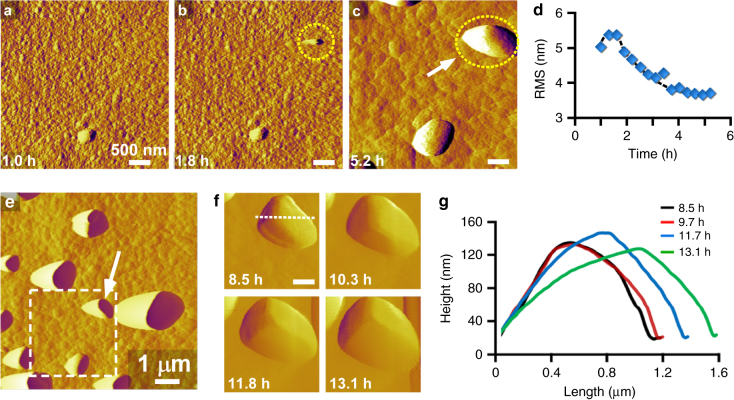
Evidence of (nearly) oriented attachment. **a–c** Time-resolved in situ AFM amplitude mode images showing the deposition of a particle from solution to the crystal surface (dashed circle). Measurements were performed in growth solution S2 heated at 45 °C. **d** Temporal change in the root mean square (RMS) roughness of a 1 × 1-μm^2^ area during continuous imaging. **e** Enlarged scan area that encompasses a region (dashed box) that was continuously imaged for 6.3 h. **f** Snapshots from Supplementary Movie 3 showing the growth of a large island (indicated by the arrow in image e). The movie taken in contact mode appears to capture island growth by classical molecule-by-molecule addition. The immediate appearance of facets on the nanoparticle suggests that these features are crystalline. **g** Graph showing the variation in height and length of a single deposit (indicated by the arrow in image c). Profiles are taken along the dashed line in image **f** as a function of time (*t* = 8.5–13.1 h). Scale bars are equal to 500 nm unless otherwise labeled

AFM measurements at higher temperature (45 °C) reveal crystal surfaces (Fig. 3a) covered with smaller islands (ca. 6 nm, Supplementary Fig. 13 and Supplementary Note 1). At these conditions, the temporal smoothening of surface features (Fig. 3b–d, and Supplementary Movie 2) is attributed to layer spreading. Indeed, there is a negligible difference in the topography of a continuously imaged area (dashed box in Fig. 3e and Supplementary Fig. 14) and its surrounding regions, which suggests that the islands are less gel-like at higher temperature, potentially due to the faster rate of crystallization. The image in Fig. 3e shows a bimodal distribution of surface feature size owing to the presence of large islands (arrow in Fig. 3e). Supplementary Movie 2 captures the generation of a large island, which occurs by the deposition of a particle from solution. These particles appear to be crystalline, suggesting that their addition to the crystal surface involves (nearly) oriented attachment, similar to processes reported for iron oxide minerals^36^. Evidence of particle crystallinity is gathered from time-resolved AFM images that reveal the immediate growth of deposits with visible facets (Fig. 3f, g and Supplementary Movie 3). Our findings indicate that the residence time of the growth solution in the AFM liquid cell (5 h) is sufficient for nucleation of nanocrystals. Reduced temperature increases the nucleation time well beyond the average liquid residence time in the AFM cell (Supplementary Fig. 1), which can explain why (nearly) oriented attachment is not observed during AFM measurements at 35 °C.

**Fig. 4:**
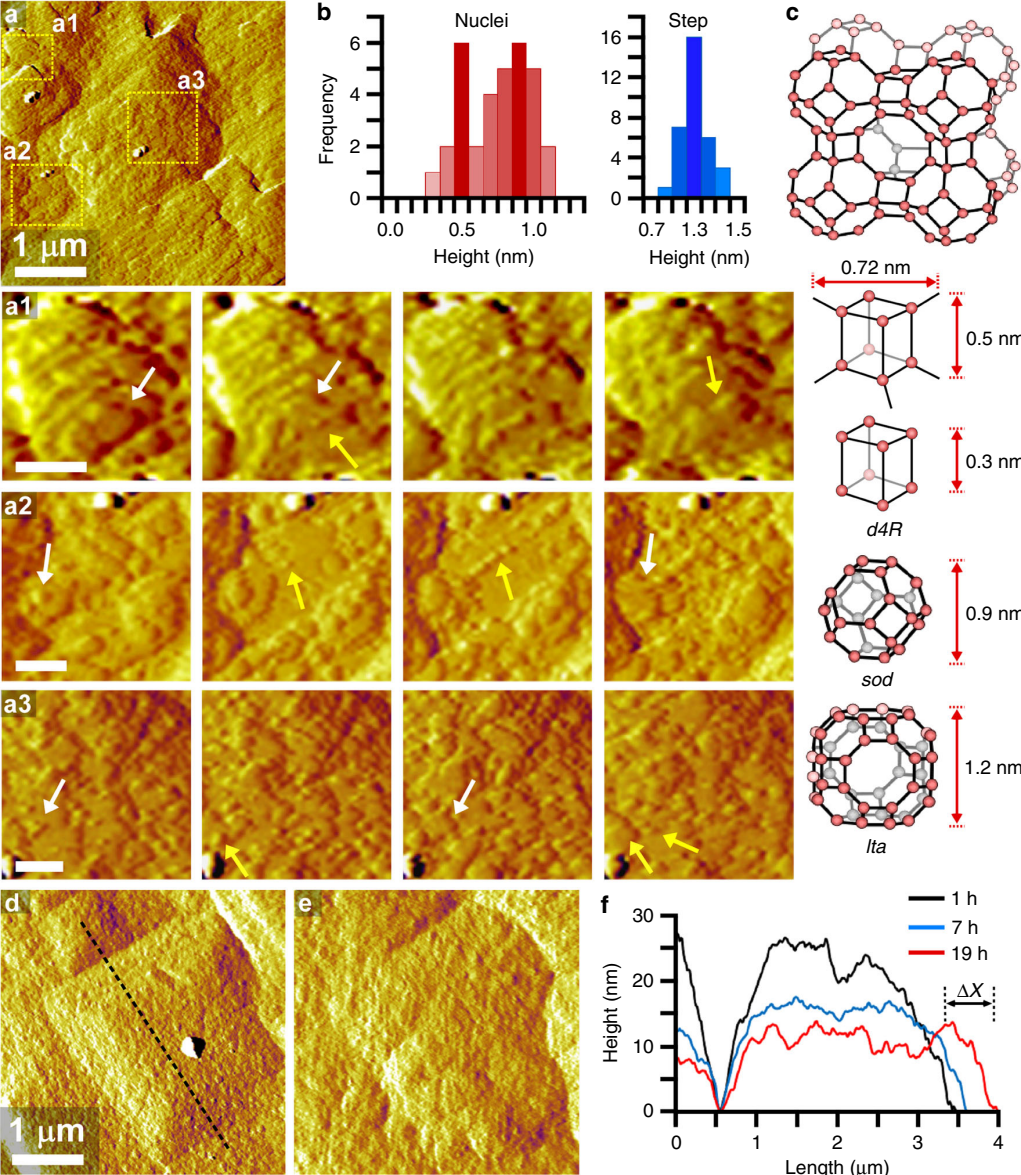
Two-dimensional layer-by-layer growth. **a** AFM amplitude mode image of a crystal surface in solution S4 (low supersaturation) at 45 °C. Snapshots from Supplementary Movie 4 in selected areas a1, a2, and a3 highlight the dynamics of surface growth (total imaging time = 30 min). White and yellow arrows depict layer growth and nuclei formation, respectively. **b** Statistical analysis of nuclei (red) and step (blue) heights measured in multiple frames of Supplementary Movie 4 (total imaging time = 90 min). **c** LTA framework and composite building units: double-four-membered ring (*d4R*), sodalite (*sod*) cage, and *lta* cage. **d**, **e** Time-resolved AFM amplitude mode images of a crystal surface growing in solution S3 at 45 °C: **d** 1 h after reaching the set point and **e** after 19 h of growth. **f** Temporal changes in the height profile along the dashed line in **d**. Scale bars are equal to 200 nm unless otherwise labeled

AFM measurements at high temperature and low supersaturation (S4) reveal a different mode of growth: two-dimensional (2D) layer nucleation and spreading. This mechanism is common in classical crystallization, but has never been observed in situ for zeolites. Other groups have inferred this mode of growth using ex situ microscopy images^37,38^ of LTA surfaces removed from saturated solutions and from in situ dissolution studies^39^ in undersaturated media; however, such approaches are incapable of fully resolving the mechanisms of layer nucleation and spreading. On the contrary, we report time-resolved images in Fig. 4 that were taken from Supplementary Movie 4 showing the dynamics of 2D growth. In these images, the original hillock is still discernable during growth, though the terraces are covered with newly generated layers as the steps advance across the surface (Supplementary Fig. 15). White arrows in high-resolution images (Fig. 4a, regions a1 to a3) depict steps that advance across the surface, whereas yellow arrows indicate the generation of 2D nuclei.

Quantitative analysis of features over multiple crystal surfaces shows two distinct populations with average heights of 0.5 and 0.9 nm (Fig. 4b). The exact molecular structure of these features cannot be established from AFM; however, it is interesting to point out that their heights are comparable to the sizes of double-four-member ring (*d4R*) and sodalite (*sod*) CBUs depicted in Fig. 4c. Park et al.^40^ proposed that high-silica LTA crystallization occurs by the formation of *sod* cages around *lta* cages. Other groups have placed more emphasis on the role of *d4R* units in LTA crystallization^20,26,27^. Studies of LTA surface terminations by transmission electron microscopy^41^ and energy minimization models^42^ suggest the possibility of both CBUs as well as other configurations (e.g., four rings). It remains to be determined from our AFM data if layer generation occurs by the direct addition of CBUs or their concerted formation at the crystal interface via the assembly of molecular species.

The comparison of newly formed nuclei and fully developed layers reveals that the latter have a much narrower distribution of step height (Fig. 4b) with an average size of 1.2 nm, equal to the *lta* cage (i.e., the length of a unit cell). As supersaturation is increased at high temperature, we observe a transition to a roughened interface (Fig. 4, d and e, and Supplementary Movie 5) that grows by a mode resembling kinetic roughening in classical theories^43,44^. The micrograph in Fig. 4d captures two neighboring hillocks that merge with increased imaging time as a result of in-plane (or lateral) layer advancement (Fig. 4f). Analysis of out-of-plane (or normal) growth is challenging due to the changing baseline. Indeed, the merging of adjacent hillocks gives the false appearance of decreasing height owing to the reduced interstitial region separating the hillocks. Evidence for kinetic roughening is provided in Supplementary Movie 6 where cycles of surface roughening and smoothening (Supplementary Fig. 16) derive from the perpetual generation and spreading of layers, respectively.

### Diverse modes of LTA crystallization

**Fig. 5:**
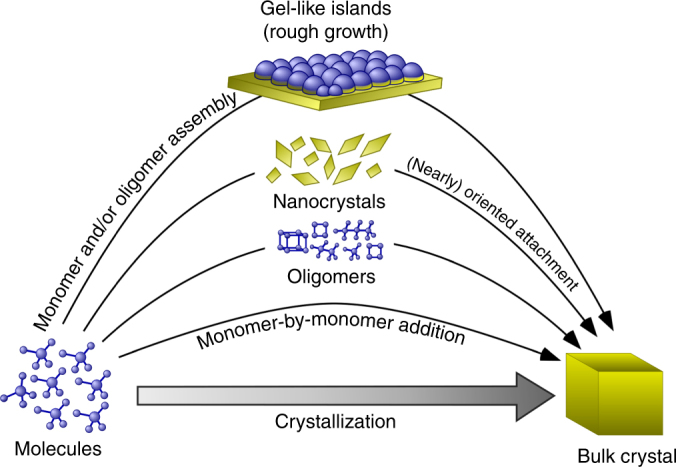
Distinct pathways of LTA crystallization. Illustrative renderings of crystal growth mechanisms for classical and nonclassical pathways. The former involves monomer-by-monomer addition that leads to growth by spirals (Fig. 1f), 2D layers (Fig. 4a), or kinetic roughening (Fig. 4d–f). In this study, we observe a unique nonclassical pathway at low temperature (*T* < 40 °C) wherein roughness derives from the formation of 3D gel-like islands (Fig. 2a–f). At higher temperatures, nucleation within the growth solution can lead to (nearly) oriented attachment of crystals to LTA surfaces (Fig. 3a–f)

Figure 5 depicts various pathways of LTA crystallization. Monomer-by-monomer addition is a classical pathway that falls within one of three modes of growth that depend upon supersaturation (Supplementary Note 2 and Supplementary Fig. 17): spiral, layer-by-layer, and roughened growth^45^. Low supersaturation (near equilibrium) is reached at the end of zeolite synthesis where ex situ AFM images of zeolite surfaces are comprised of hillocks. Sacco et al.^21^ previously showed the presence of spiral dislocations on the upper terrace of hillocks. Higher supersaturation results in layer-by-layer growth, which occurs by 2D generation and spreading of layers via the addition of monomers and/or oligomers. At high supersaturation, classical theories posit rough growth as a result of low energetic barriers for nucleation, whereby the solute can bind at all possible sites, allowing for the rapid formation of nuclei with critical sizes less than those required for layered growth. This phenomenon, commonly referred to as kinetic roughening, is observed for LTA growth at high temperature; however, the roughness observed at low temperature derives from a new type of surface feature that has not previously been reported. Notably, the formation of gel-like islands from molecularly dispersed solute constitutes a unique mode of growth among the reported cases of nonclassical crystallization. Measurements of LTA crystallization also reveal the first direct observation of zeolite growth by the (nearly) oriented attachment of crystals, which is an established nonclassical pathway in the formation of other types of minerals^4^.

These findings offer new insight into the complex processes of sol–gel chemistry that are integral to zeolite formation and that are potentially applicable to other minerals that crystallize via similar routes. Elucidating the mechanism(s) of zeolite crystallization is challenging owing to the multitude of species that serve as putative growth units, as well as the lack of in situ techniques that are capable of resolving growth processes at sufficient spatiotemporal resolution. To this end, solvothermal AFM is a suitable tool for capturing time-resolved events of surface growth. Beyond identifying the modes of LTA growth, we have used these observations to establish that the choice of synthesis conditions governs distinct regimes of zeolite growth, and therefore, can be integral to rational design. Moreover, our findings reconcile disparate hypotheses of LTA growth wherein gel-to-crystal transitions have for many years been assumed to play a key role in zeolite crystallization. Given that the conditions selected for this study are similar to a wide range of zeolite framework types, the pathways of crystal growth for LTA may prove to be representative of other related materials.

## Methods

### Chemicals for zeolite synthesis and growth experiments

The following chemicals were used as reagents: sodium hydroxide (98% pellets, MACRON Fine Chemicals), sodium aluminate (Al_2_O_3_·Na_2_O or NaAlO_2_, 54.41% Al_2_O_3_, and 41.02% Na_2_O, Alfa Aesar), Ludox AS-40 (40%, Sigma Aldrich), tetraethylorthosilicate (TEOS, 98%, Sigma Aldrich, St. Louis, MO), and triethanolamine (TEA, 100%, J.T. Baker). Deionized (DI) water was prepared using an Aqua Solutions RODI-C-12A purification system (18.2 MΩ). All reagents were used as received without further purification.

### Synthesis of zeolite substrates for scanning probe microscopy

Cubic crystals of zeolite LTA were synthesized using a previously reported protocol^20^. The growth solution was prepared by mixing the required quantity of all components to yield a solution with molar composition 1.23 SiO_2_:1 Al_2_O_3_:2.71 Na_2_O:5.5 TEA:288 H_2_O. To obtain a 15-g mixture, 0.28 g of NaOH was added to 11.80 g of DI water and mixed in a polypropylene (PP) container. Thereafter, 0.43 g of sodium aluminate was added to this mixture and stirred for 30 min to obtain a clear solution, followed by the addition of 1.89 g of TEA. To this mixture 0.60 g of TEOS was added. The solution was then aged at room temperature for 4 h while stirring. The PP container was heated in a Thermo-Fisher Precision Premium 3050 Series gravity oven at 85 °C for 11 days and was quenched to room temperature. The precipitate was isolated using a 0.45-μm GHP filter (Pall Corporation) and washed multiple times with DI water.

### Preparation of zeolite growth solutions

The growth solution for in situ AFM experiments was prepared from a 60-g mixture with molar composition 1 SiO_2_:0.87 Al_2_O_3_:11.2 NaOH:190.6 H_2_O. In a PP container, 5.47 g of NaOH was added to 49.86 g of degassed DI water. Thereafter, 2.44 g of sodium aluminate was added and the resulting mixture was stirred for 30 min. To this solution, 2.24 g of Ludox AS-40 was added and the mixture was left aging at room temperature for 24 h while stirring. The growth solution was heated in a Thermo-Fisher Precision Premium 3050 Series gravity oven at 65 °C for various times and quenched in an ice bath. The precipitate was removed after centrifuging the solution at 13,000 rpm for 45 min in a Beckman Coulter Avanti J-E instrument. The supernatant was decanted and filtered twice, using a 25-mm syringe filter fitted with a 0.45-μm nylon membrane (VWR International). The resulting clear supernatant was used as a growth solution for in situ AFM measurements. The solutions (labeled in Fig. 1c) are referred to as S1, S2, S3, etc., where “S” refers to supernatant and the number represents the total hours of preheating at 65 °C.

### In situ atomic force microscopy

All AFM measurements were performed on an Asylum Research MFP-3D-SA instrument (Santa Barbara, CA) equipped with a custom-designed liquid sample cell for imaging under solvothermal conditions. A detailed description of the liquid cell is provided in a previous publication^9^. Cubic LTA crystals prepared as substrates for AFM were firmly placed on a 15-mm specimen disk (Ted Pella, Inc.) using quickset Loctite epoxy (Henkel Corporation) that was cured in an oven at 60 °C for 1 h. The sample was then removed from the oven and cooled to room temperature in air. The sample was rinsed with DI water to remove loosely bound crystals, and dried under inert Ar gas to remove dust. The sample was then placed in a closed AFM liquid cell (total volume ≈ 3 ml). AFM images were collected using a Cr/Au-coated silicon nitride cantilever (Olympus RC800PB) with a spring constant of 0.82 N m^−1^. The LTA crystal substrate was first scanned in air to locate a desired imaging area. The growth solution was then introduced into the AFM cell by a syringe and the sample was left to equilibrate with the solution at room temperature for ca. 30 min. The temperature was then ramped to a predetermined set point at a rate of 1 °C min^−1^. Growth solution was continuously supplied to the liquid cell using a syringe pump (Razel Scientific Instruments, Model R100-E) at a rate of 0.9 cm^3^ h^−1^. Once the sample cell reached the set point temperature, the liquid flow rate was reduced to 0.6 cm^3^ h^−1^, which equates to an average residence time of 5 h. The sample cell was allowed to equilibrate for 0.5–1 h before imaging. AFM images were collected in tapping mode (to minimize tip–substrate contact) at a scan rate of 1.2 Hz and 256 lines per scan. Select areas were periodically imaged in contact mode when it was necessary to resolve large surface features.

### Chemical force microscopy

CFM has proven to be a useful technique for measuring the unbinding force between AFM tips and sample interfaces^34,46^. AFM tips can be functionalized with an array of chemical moieties^47,48^ or larger macromolecules (e.g., DNA or proteins)^49–55^. The information gleaned from these studies ranges from thermodynamics of tip–substrate bond breakage^56–60^ to the dynamics of unraveling segments of macromolecules^54,61^. Recently, CFM has been used to investigate elastic substrates, such as lipids, surfactants, and thin films^35,62–66^. These studies have shown that the elastic-like character of substrates can yield nonlinear approach–retraction curves.

We used the MFP-3D-SA instrument to measure the unbinding force between an AFM tip and LTA crystal surfaces. All measurements were carried out using a non-functionalized Cr–Au-coated silicon nitride cantilever (Olympus RC800PB) with a spring constant of 0.15 N m^−1^. The cantilever was calibrated in air to verify the spring constant using an algorithm provided by the vendor. Growth solution S2 was introduced into the AFM liquid cell and was allowed to equilibrate for 2 h at room temperature. We first imaged as-synthesized LTA crystal surfaces in contact mode to locate regions for CFM measurements. Force curves at room temperature correspond to pristine surfaces (see Supplementary Fig. 9). We selected a tip speed of 1 μm s^−1^ and trigger points of 2, 5, and 10 nN. A dwell time of 1 s was used for all measurements to promote tip–substrate interactions (note that the dwell time refers to a period when the tip is held in direct contact with the surface after engaging). We analyzed surface areas of 4 × 4 μm^2^ to gather more than 250 data points for statistical analysis. After measuring pristine surfaces, the temperature was ramped to 35 °C at a rate of 1 °C min^−1^ while maintaining constant flow of growth solution at 0.9 cm^3^ h^−1^. Once reaching the set point, the flow of growth solution was reduced to 0.6 cm^3^ h^−1^ and the system was equilibrated for 30 min. LTA crystal substrates were allowed to grow for an additional 4 h. The surface was imaged to confirm the presence of gel-like islands (i.e., rough surfaces). The temperature was then ramped down to room temperature and the AFM sample holder was removed and washed with DI water. The sample was again placed in the liquid cell with a new cantilever that was calibrated in air. Fresh growth solution (S2) was introduced into the liquid cell. After 2-h equilibration at room temperature, CFM measurements were performed as described above (see Supplementary Fig. 9).

### Small-angle X-ray scattering

SAXS measurements were performed to check for the presence of (alumino)silicate nanoparticle precursors in the supernatant growth solutions used for in situ AFM studies. Clear growth solutions, prepared using the aforementioned protocol, were injected into a clean sample holder (1.5-mm quartz capillary cell). Scattering patterns were collected under vacuum at 25 °C for 30 min using a Rigaku S-MAX3000 instrument (CuKα radiation *λ* = 1.54 Å; *q* = 0.008–0.24 Å^−1^). Calibrations of the scattering vector *q* and beam center were performed on raw data using the SAXS gui software provided by Rigaku and a reference pattern from a AgBeh standard that was collected for 500 s. A background (DI water) was subtracted from each sample. Normalized SAXS patterns were compared to those reported in previous studies of silicalite-1 precursors^12,13,67–69^ to check for the presence of nanoparticles in LTA growth solutions.

### Dynamic light scattering

DLS was performed on a Brookhaven Instruments BI-200SM machine equipped with a TurboCorr Digital Correlator, a red HeNe laser diode (35 mW and 637 nm), and a decalin vat fitted with a filter to remove dust. Supernatant solutions (described previously) were used for particle size measurements. At least three measurements were performed per sample using a scattering angle of 90°. Autocorrelation functions were collected over 2 min and the data were evaluated using the method of cumulants to extract particle size assuming a refractive index of pure water and a viscosity that was measured using a calibrated CUC-25 Cannon Ubbelohde viscometer (9721-K50, kinematic viscosity range 0.4−2.0 mm^2^ s^−1^). In order to perform in situ DLS measurements, clear growth solutions were transferred to 20-ml disposable scintillation vials that were placed inside the decalin vat. Measurements were first taken at a temperature of 25 °C, which was maintained using a Polyscience digital temperature controller. Thereafter, the temperature was ramped to 45 °C at the rate of 1 °C min^−1^. After reaching the set point temperature, measurements were taken periodically to track the average particle size. Time-elapsed measurements of growth solutions S1 and S2 are shown in Supplementary Figure 1. Counts were too low for measurements of S3 and S4 growth solutions (even after 24 h of heating).

### Scanning electron microscopy

Scanning electron micrographs were obtained with a FEI 235 dual-beam (focused ion-beam) system operated at 15 kV and a 5-mm working distance. All SEM samples were coated with a thin carbon layer (ca. 20 nm) prior to imaging.

### Energy dispersive X-ray spectroscopy

EDX analysis was performed using a JEOL JSM 6330 F field-emission SEM operated at 12 kV and 15-mm working distance.

### X-ray photoelectron spectroscopy

XPS spectra were collected from a Physical Electronics Model 5700 XPS instrument. A monochromatic Al_Kα_ X-ray source (1486.6 eV) was used with the power at 350 W. All spectra were obtained once reaching a vacuum of 5 × 10^−9^ torr or better.

### Powder X-ray diffraction

Powder XRD patterns of as-synthesized zeolite samples were collected on a Siemens D5000 X-ray diffractometer using a CuKα source (40 kV, 30 mA). The LTA framework was confirmed using a reference pattern provided by the International Zeolite Association Structure Database^70^.

### Data availability

The data that support the findings of this study are available from the corresponding author on reasonable request.

## Electronic supplementary material

Supplementary Information

Description of Additional Supplementary Files

Supplementary Movie 1

Supplementary Movie 2

Supplementary Movie 3

Supplementary Movie 4

Supplementary Movie 5

Supplementary Movie 6

## References

[1] Habraken, W. et al. Ion-association complexes unite classical and non-classical theories for the biomimetic nucleation of calcium phosphate. *Nat. Commun*. **4**, 1507 (2013). 10.1038/ncomms2490 23422675

[2] Zhang L (2015). Platinum-based nanocages with subnanometer-thick walls and well-defined, controllable facets. Science.

[3] Zheng JP (2009). From molecular to macroscopic via the rational design of a self-assembled 3D DNA crystal. Nature.

[4] De Yoreo, J. J. et al. Crystallization by particle attachment in synthetic, biogenic, and geologic environments. *Science* **349**, 6760 (2015). 10.1126/science.aaa6760 26228157

[5] Nielsen MH, Aloni S, De Yoreo JJ (2014). In situ TEM imaging of CaCO3 nucleation reveals coexistence of direct and indirect pathways. Science.

[6] Liao HG, Cui LK, Whitelam S, Zheng HM (2012). Real-time imaging of Pt3Fe nanorod growth in solution. Science.

[7] Banfield JF, Welch SA, Zhang HZ, Ebert TT, Penn RL (2000). Aggregation-based crystal growth and microstructure development in natural iron oxyhydroxide biomineralization products. Science.

[8] Aerts A, Kirschhock CE, Martens JA (2010). Methods for in situ spectroscopic probing of the synthesis of a zeolite. Chem. Soc. Rev..

[9] Lupulescu AI, Rimer JD (2014). In Situ imaging of silicalite-1 surface growth reveals the mechanism of crystallization. Science.

[10] Shete M (2017). Nanoscale control of homoepitaxial growth on a two-dimensional zeolite. Angew. Chem. Int. Ed..

[11] Kumar M, Luo H, Roman-Leshkov Y, Rimer JD (2015). SSZ-13 crystallization by particle attachment and deterministic pathways to crystal size control. J. Am. Chem. Soc..

[12] Davis TM (2006). Mechanistic principles of nanoparticle evolution to zeolite crystals. Nat. Mater..

[13] Kumar S, Davis TM, Ramanan H, Penn RL, Tsapatsis M (2007). Aggregative growth of silicalite-1. J. Phys. Chem. B.

[14] Mintova S, Olson NH, Bein T (1999). Electron microscopy reveals the nucleation mechanism of zeolite Y from precursor colloids. Angew. Chem. Int. Ed..

[15] Ren N (2012). Unusual pathway of crystallization of zeolite ZSM-5 in a heterogeneous system: phenomenology and starting considerations. Chem. Mat..

[16] Corma A, Rey F, Rius J, Sabater MJ, Valencia S (2004). Supramolecular self-assembled molecules as organic directing agent for synthesis of zeolites. Nature.

[17] Ryu T (2017). Fully copper-exchanged high-silica LTA zeolites as unrivaled hydrothermally stable NH3-SCR Catalysts. Angew. Chem. Int. Ed..

[18] Smaihi, M., Barida, O. & Valtchev, V. Investigation of the crystallization stages of LTA-type zeolite by complementary characterization techniqnes. *Eur. J. Inorg. Chem*. **2003**, 4370–4377, (2003).

[19] Fan W (2007). Effects of silicon sources on the formation of nanosized LTA: an in situ small angle X-ray scattering and wide angle X-ray scattering study. Micro. Mesopor. Mater..

[20] Sugiyama S (1999). AFM observation of double 4-rings on zeolite LTA crystals surface. Micro. Mesopor. Mater..

[21] Dumrul S, Bazzana S, Warzywoda J, Biederman RR, Sacco A (2002). Imaging of crystal growth-induced fine surface features in zeolite A by atomic force microscopy. Micro. Mesopor. Mater..

[22] Yang CS, Mora-Fonz JM, Catlow CRA (2013). Modeling the nucleation of zeolite A. J. Phys. Chem. C..

[23] Van Speybroeck V (2015). Advances in theory and their application within the field of zeolite chemistry. Chem. Soc. Rev..

[24] Mintova S, Olson NH, Valtchev V, Bein T (1999). Mechanism of zeolite A nanocrystal growth from colloids at room temperature. Science.

[25] Valtchev VP, Bozhilov KN (2005). Evidences for zeolite nucleation at the solid-liquid interface of gel cavities. J. Am. Chem. Soc..

[26] Ren L (2011). UV-Raman and NMR spectroscopic studies on the crystallization of zeolite A and a new synthetic route. Chemistry.

[27] Xiao YC (2017). Mechanism on solvent-free crystallization of NaA zeolite. Micro. Mesopor. Mater..

[28] Maldonado M, Oleksiak MD, Chinta S, Rimer JD (2013). Controlling crystal polymorphism in organic-free synthesis of Na-Zeolites. J. Am. Chem. Soc..

[29] Oleksiak MD, Soltis JA, Conato MT, Penn RL, Rimer JD (2016). Nucleation of FAU and LTA zeolites from heterogeneous aluminosilicate precursors. Chem. Mat..

[30] Fan W (2006). In situ observation of homogeneous nucleation of nanosized zeolite A. Phys. Chem. Chem. Phys..

[31] Kragten DD (2003). Structure of the silica phase extracted from silica/(TPA)OH solutions containing nanoparticles. J. Phys. Chem. B..

[32] de Moor P, Beelen TPM, Komanschek BU, Diat O, van Santen RA (1997). In situ investigation of Si-TPA-MFI crystallization using (ultra-) small- and wide-angle X-ray scattering. J. Phys. Chem. B..

[33] Watson, J. N., Iton, L. E. & White, J. W. In situ observation of the growth of silicalite nuclei by small-angle X-ray and neutron scattering. *Chem. Commun*. **0**, 2767–2768, (1996).

[34] Butt HJ, Cappella B, Kappl M (2005). Force measurements with the atomic force microscope: technique, interpretation and applications. Surf. Sci. Rep..

[35] Pera I, Stark R, Kappl M, Butt HJ, Benfenati F (2004). Using the atomic force microscope to study the interaction between two solid supported lipid bilayers and the influence of synapsin I. Biophys. J..

[36] Li DS (2012). Direction-specific interactions control crystal growth by oriented attachment. Science.

[37] Cubillas P (2011). AFM and HRSEM invesitigation of zeolite A crystal growth. Part 1: in the absence of organic additives. J. Phys. Chem. C.

[38] Agger JR, Pervaiz N, Cheetham AK, Anderson MW (1998). Crystallization in zeolite A studied by atomic force microscopy. J. Am. Chem. Soc..

[39] Itzel Meza L, Anderson MW, Slater B, Agger JR (2008). In situ atomic force microscopy of zeolite A dissolution. Phys. Chem. Chem. Phys..

[40] Park MB (2013). Formation pathway for LTA zeolite crystals synthesized via a charge density mismatch approach. J. Am. Chem. Soc..

[41] Wakihara T, Sasaki Y, Kato H, Ikuhara Y, Okubo T (2005). Investigation of the surface structure of zeolite A. Phys. Chem. Chem. Phys..

[42] Slater B, Titiloye JO, Higgins FM, Parker SC (2001). Atomistic simulation of zeolite surfaces. Curr. Opin. Solid State Mat. Sci..

[43] Cuppen HM, Meekes H, van Enckevort WJP, Vissers GWM, Vlieg E (2004). Kinetic roughening of Kossel and non-Kossel steps. Surf. Sci..

[44] Halpinhealy T, Zhang YC (1995). Kinetic roughening phenomena, stochastic growth directed polymers and all that. Aspects of multidisciplinary statistical mechanics. Phys. Rep..

[45] Tilbury CJ, Doherty MF (2017). Modeling layered crystal growth at increasing supersaturation by connecting growth regimes. AIChE J..

[46] Noy A, Vezenov DV, Lieber CM (1997). Chemical force microscopy. Annu. Rev. Mater. Sci..

[47] Frisbie CD, Rozsnyai LF, Noy A, Wrighton MS, Lieber CM (1994). Functional-group imaging by chemical force microscopy. Science.

[48] Noy A (2006). Chemical force microscopy of chemical and biological interactions. Surf. Interface Anal..

[49] Rief M, Gautel M, Oesterhelt F, Fernandez JM, Gaub HE (1997). Reversible unfolding of individual titin immunoglobulin domains by AFM. Science.

[50] Bao G, Suresh S (2003). Cell and molecular mechanics of biological materials. Nat. Mater..

[51] Strunz T, Oroszlan K, Schafer R, Guntherodt HJ (1999). Dynamic force spectroscopy of single DNA molecules. Proc. Natl Acad. Sci. USA.

[52] Evans E (2001). Probing the relation between force - Lifetime - and chemistry in single molecular bonds. Annu. Rev. Biophys. Biomol. Struct..

[53] Carrion-Vazquez M (1999). Mechanical and chemical unfolding of a single protein: a comparison. Proc. Natl Acad. Sci. USA.

[54] del Rio A (2009). Stretching single talin rod molecules activates vinculin binding. Science.

[55] Wong SS, Joselevich E, Woolley AT, Cheung CL, Lieber CM (1998). Covalently functionalized nanotubes as nanometre-sized probes in chemistry and biology. Nature.

[56] Noy A, Friddle RW (2013). Practical single molecule force spectroscopy: how to determine fundamental thermodynamic parameters of intermolecular bonds with an atomic force microscope. Methods.

[57] Friddle RW, Noy A, De Yoreo JJ (2012). Interpreting the widespread nonlinear force spectra of intermolecular bonds. Proc. Natl Acad. Sci. USA.

[58] Friddle RW (2011). Single-molecule determination of the face-specific adsorption of amelogenin’s C-terminus on Hydroxyapatite. Angew. Chem. Int. Ed..

[59] Noy A, Zepeda S, Orme CA, Yeh Y, De Yoreo JJ (2003). Entropic barriers in nanoscale adhesion studied by variable temperature chemical force microscopy. J. Am. Chem. Soc..

[60] Friddle RW, Podsiadlo P, Artyukhin AB, Noy A (2008). Near-equilibrium chemical force microscopy. J. Phys. Chem. C..

[61] Rief M, Fernandez JM, Gaub HE (1998). Elastically coupled two-level systems as a model for biopolymer extensibility. Phys. Rev. Lett..

[62] Garcia-Manyes S, Sanz F (2010). Nanomechanics of lipid bilayers by force spectroscopy with AFM: a perspective. Biochim. Biophys. Acta.

[63] Garcia-Manyes S, Redondo-Morata L, Oncins G, Sanz F (2010). Nanomechanics of lipid bilayers: heads or tails?. J. Am. Chem. Soc..

[64] Ong YL, Razatos A, Georgiou G, Sharma MM (1999). Adhesion forces between E-coli bacteria and biomaterial surfaces. Langmuir.

[65] Manne S, Cleveland JP, Gaub HE, Stucky GD, Hansma PK (1994). Direct visualization of surfactant hemimicelles by force microscopy of the electrical double layer. Langmuir.

[66] Johnson SB, Drummond CJ, Scales PJ, Nishimura S (1995). Comparison of techniques for measuring the electrical double -layer properties of surfaces in aqueous-solution-Hexadecyltrimethylammonium bromide self-assembly structures as a model system. Langmuir.

[67] Fedeyko JM, Rimer JD, Lobo RF, Vlachos DG (2004). Spontaneous formation of silica nanoparticles in basic solutions of small tetraalkylammonium cations. J. Phys. Chem. B.

[68] Rimer JD, Vlachos DG, Lobo RF (2005). Evolution of self-assembled silica-tetrapropylammonium nanoparticles at elevated temperatures. J. Phys. Chem. B.

[69] Aerts A (2007). Combined NMR, SAXS, and DLS study of concentrated clear solutions used in silicalite-1 zeolite synthesis. Chem. Mat..

[70] International Zeolite Association. http://www.iza-structure.org/. Accessed 22 May 2017.

