# Supplementary material for: Transient modes of zeolite surface growth from 3D gel-like islands to 2D single layers
Source: Nat Commun. 2018 May 29;9:2129. doi: 10.1038/s41467-018-04296-4 (PMC5974312; doi:10.1038/s41467-018-04296-4)
Supplement: Supplementary file 1 — Supplementary Information [file 41467_2018_4296_MOESM1_ESM.pdf]

Transient modes of zeolite surface growth from 3D gel-like islands to 2D single layers

Kumar *et. al*.

Supplementary Note 1. Statistical analysis of feature heights in AFM images.

Histograms of feature heights in AFM images were generated using the Igor Pro software provided by the vendor. The software plots the height distribution of individual pixels on AFM height images against the baseline of an average height. Standard deviations were calculated from Gaussian fits to each distribution of feature heights. We used the image analysis tool to obtain Gaussian fits to each data set. Negative values of height can occur given that the average height is used as a reference.

Supplementary Note 2. Classical theories of crystal growth.

Mechanisms of crystallization tend to involve the nucleation and spreading of layers via monomer-by-monomer addition. This mode of growth has been confirmed for numerous crystals[1]. There are various physicochemical parameters, such as supersaturation and temperature, which directly impact growth pathway(s). Supplementary Fig. 17 shows three distinct regimes of growth that are broadly differentiated on the basis of the relative supersaturation σ,

$$\sigma = \frac{C}{C_o} - 1 \qquad (1)$$

$$\Delta\mu = kTln(1 + \sigma) \equiv kT\sigma \qquad (2)$$

where k is the Boltzmann constant, T is temperature, $\Delta\mu$ is the change in chemical potential per molecule, C is the solute concentration, and C_o is the equilibrium concentration (or solubility).

At low σ (Supplementary Fig. 17, Region I), surfaces grow by the direct addition of solute to defect sites (e.g., screw dislocations[2]), resulting in the formation of hillocks. AFM has proven to be a useful technique for capturing the presence and growth of such features on the surfaces of inorganic[3,4] and organic crystals[5-7]. The growth of a crystal surface follows different dynamics depending on the limitations of solute addition. The Damköhler number (*D*a) is often used to differentiate the rate limiting steps. For instance, when Da << 1 the growth rate is limited by surface integration, and when Da >>1 the growth rate it is limited by bulk transport. In the kinetic regime, the normal growth rate *R* of a surface[4,8] exhibits a parabolic relationship with σ:

$$R = C''\sigma^2 \qquad (3)$$

The rate constant C" is a function of several parameters,

$$C'' = \frac{nkT}{19\chi a} \beta_{st} C_0 v_c \quad (4)$$

where a is the step height, β_{st} is the kinetic coefficient of step growth, v_c is the volume of a single building unit, and n is the number of dislocations. The parameter χ is given by

$$\chi = (1 + \frac{D_S}{\lambda_s \beta_{st}})^{-1} \quad (5)$$

where λ_s is the mean distance covered by adatoms during their lifetime, and D_s is the surface diffusion coefficient.

At higher supersaturation (Supplementary Fig. 17, Region II), the rate of 2D nucleation becomes sufficiently high to compete with spiral growth, and there is a crossover to the regime where crystallization occurs by 2D layer nucleation and spreading[9]. This mode of growth has been observed for a wide range of materials that include metals[10], inorganics[11], and organics[12]. Nuclei can form on defect free terraces and generate a new layer that advances by monomer addition to steps sites (e.g., kinks). The rate of 2D nucleation determines the mode of growth. At low nucleation rate, the layer extends across the entire crystal surface prior to a new nucleation event. This phenomenon, which is typically referred to as monolayer growth, is more pronounced on small crystal faces at low supersaturation. In this regime, the nucleation rate varies exponentially with supersaturation while the rate of step growth follows a linear trend. Conversely, the formation of new nuclei before the completion of the underlying layer gives rise to multilayer growth[13]. This regime can be subdivided into two categories depending on the growth area and nucleating area. If the growth area is larger than the nucleating area, the layer emanating from a sparse population of nucleating centers will start merging, and the growth rate of the crystal surface is dominated by the step velocity. However, if the rate of nucleation is high enough that steps merge quickly due to a large population of nuclei, it is referred to as the polynuclear regime. Our observations suggest that LTA surfaces exhibit multilayer growth, $R_{2D,\ ML}$, via a birth and spread model:

$$R_{2D,ML} = \omega(\Delta\mu)^{5/6} \exp(-\frac{\Delta G_c}{3kT}) \quad (6)$$

The term ΔG_c (α $1/\Delta\mu$) is the free energy barrier for 2D nucleation. The constant ω is expressed as

$$\omega = [\frac{K_1}{\sqrt{\sigma}}(\beta_{st} C_0 v_c)^2]^{1/3} \quad (7)$$

Where K_1 is a pre-exponential term for 2D nucleation in solution.

As the supersaturation increases further, classical theory posits a transition from smooth (layered) to rough growth. This phenomenon, which is commonly referred to as kinetic roughening, has been observed for various crystalline materials. The transition from smooth to rough growth can be controlled by supersaturation and/or temperature[14-18]. During kinetic roughening, there is a loss of faceting on crystal surfaces owing to rounded step edges, which can have a concomitant effect on the bulk morphology of crystals[19-21]. Based on the Gibbs-Thompson inverse correlation between a critical radius and supersaturation, the size of a nucleus in this regime is generally

smaller than the critical nucleus size for 2D generation of islands at higher supersaturation. This is attributed to a negligible energetic barrier[22] for solute attachment to crystal surfaces, which renders molecules (i.e., monomers) or small clusters thereof as viable nuclei. Under conditions when barriers to monomer addition are small, there is a high density of nuclei on the surface wherein the average distance between layers is small (e.g., interatomic distances). This, in turn, leads to highly rough surfaces when monomers can attach at all possible binding sites on crystal surfaces.

Various theoretical models[23-26] based on either known or estimated thermodynamic parameters[27,28] have been used to modify the original growth models of Burton, Cabrera, and Frank[29] to account for kinetic roughening. Measurements of crystal growth in the kinetic roughening regime (Supplementary Fig. 17, Region III) reveal that the normal growth rate of crystal surfaces varies linearly with increasing supersaturation[30], expressed as

$$R_{KR} \sim C_{KR} C_o \sigma \qquad (8)$$

where C_{KR} is the kinetic rate constant for kinetic roughening (labelled "*KR*"). In our study, we show a unique form of roughening at low temperature owing to the formation of gel-like islands. Our findings indicate that it is possible for 3D nucleation to occur in a cohesive region when there is partial wetting of the surface by solute. This regime requires higher supersaturation than that of 2D nucleation. The fact that we observe gel-like islands in LTA surface growth at sufficiently high σ suggests a transition from an adhesive region (2D nucleation) to a cohesive region (3D nucleation).

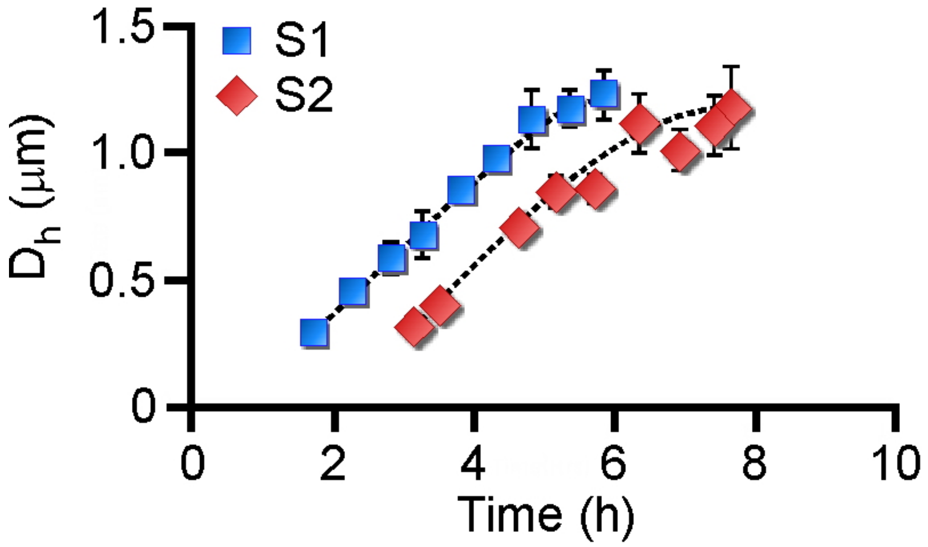

Supplementary Figure 1│Time-elapsed particle evolution. *In situ* DLS measurements of supernatant solutions S1 (blue squares) and S2 (red diamonds) heated at 45°C as a function of time. Solutions at initial times do not contain any detectable particles, consistent with SAXS measurements in figure 1d. These growth curves show an induction period that increases with decreased supersaturation. During periods of growth, there is a linear increase in the average hydrodynamic diameter D_h of crystals. The particle size eventually plateaus at longer times as solutions approach equilibrium. Note that the scattering intensity from supernatant solutions S3 and S4 was insufficient for DLS measurements. Also, similar measurements at 35°C (not shown) over a 12 h period showed no sign of crystal nucleation, indicating a much longer induction time at lower temperature, as expected. The hydrodynamic diameters reported here account for the kinematic viscosity of the growth solution, which was *ca.* 1.13 mm^2s^{-1} for both S1 and S2.

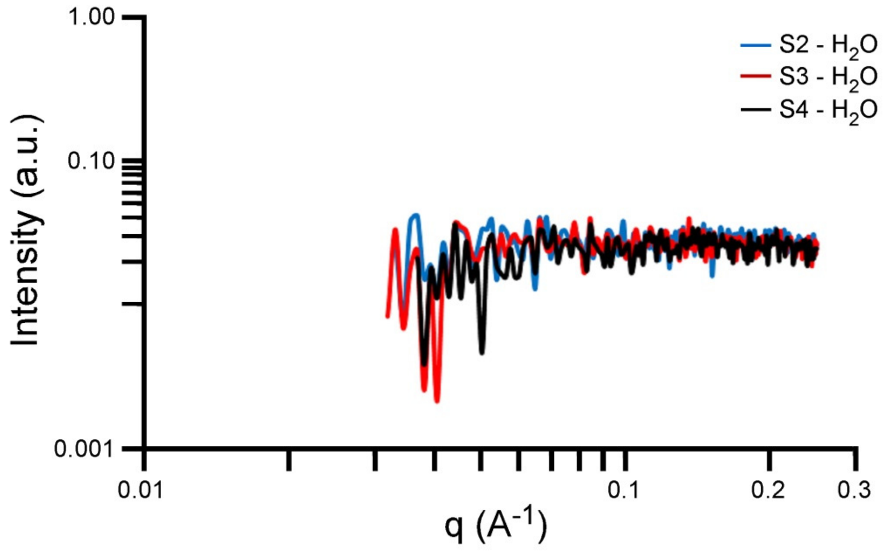

Supplementary Figure 2 | Determination of particle in growth solution. Small-angle X-ray scattering patterns of supernatant solutions S2 (blue), S3 (red), and S4 (black) with subtracted background patterns (water). The background subtracted patterns exhibit no trace of nanoparticles for all three growth solutions. It is feasible that the absence of particles may be attributed to the limitations of SAXS analysis, such as low particle number density and/or insufficient solvent-particle contrast.

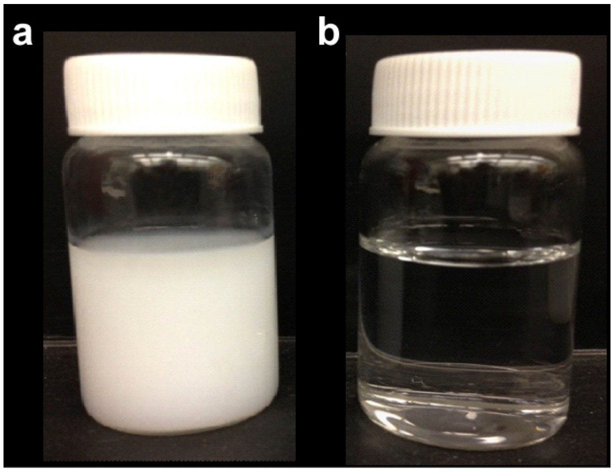

Supplementary Figure 3 | Growth solution before and after filtration. a. Growth solutions prepared for *in situ* AFM measurements prior to filtration are opaque. **b.** The same growth solution after centrifugation at 13,000 rpm for 45 min and then filtering twice. The resulting clear solution is representative of the growth solutions supplied to the liquid sample cell for AFM and CFM measurements.

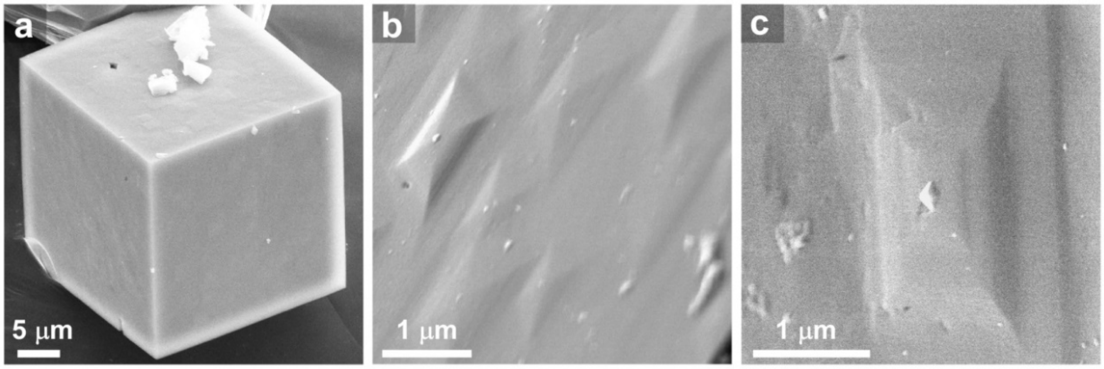

Supplementary Figure 4│ Zeolite A seed crystal. a. Scanning electron micrograph of a representative LTA crystal used as a substrate for *in situ* AFM measurements. **b and c.** Corresponding high resolution SEM images of crystal surfaces showing the presence of hillocks.

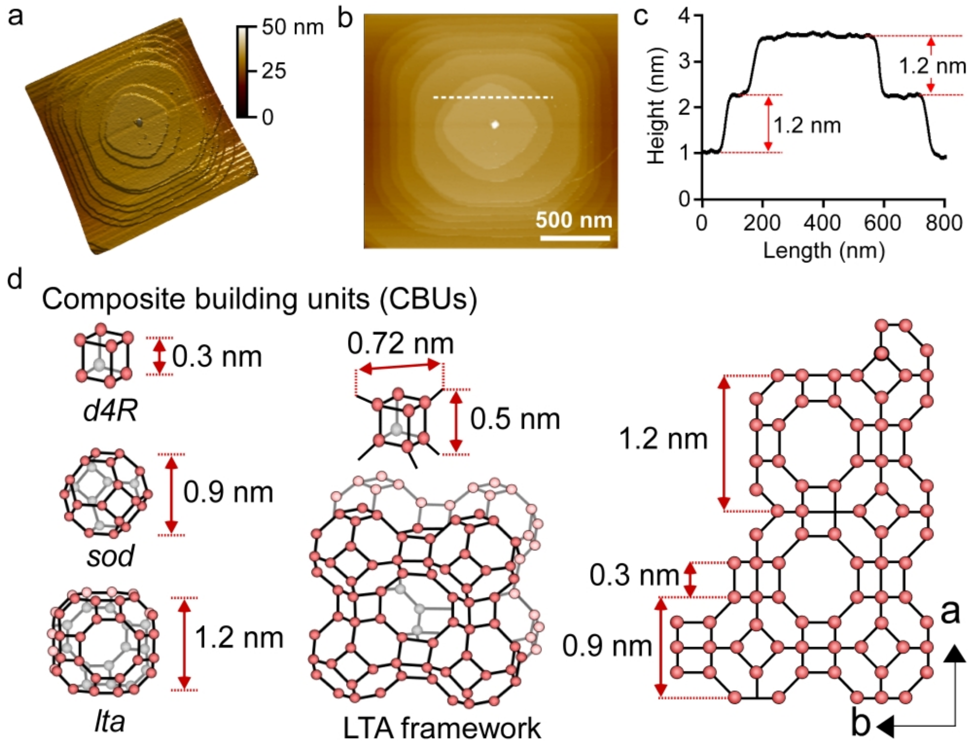

Supplementary Figure 5│Surface architecture of a LTA crystal substrate. a and b. A 3-dimensional and corresponding 2-dimensional height mode scan of a single hillock on the surface of a LTA crystal. This image is representative of hillocks observed on numerous LTA samples. **c.** Height profile along the dashed white line labeled in image b. The average heights of steps are 1.2 nm, which corresponds to the unit cell dimension of LTA crystals. In many cases we also observe step bunches (i.e., heights corresponding to multiple unit cells). **d.** Schematics of composite building units (CBUs) have dimensions that are consistent with the newly generated islands and advancing layers on LTA crystal surfaces, as observed in AFM images. The schematic on the right is an idealized arrangement of a crystal lattice that gives rise to a distribution of step heights (depicted along the *c*-direction).

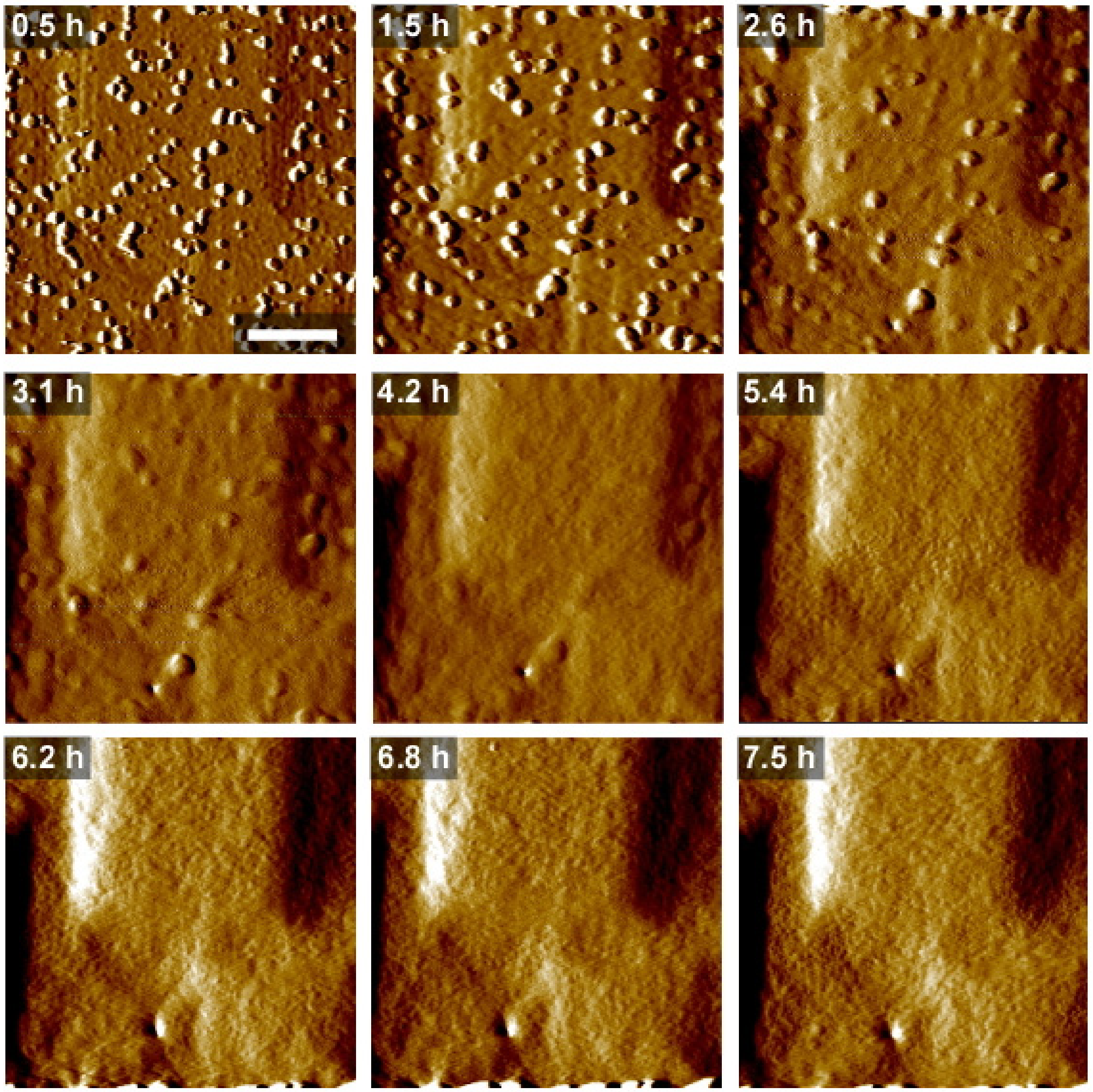

Supplementary Figure 6 | Surface evolution at low temperature and high saturation. Time-resolved AFM amplitude mode images of LTA surface growth at 35°C in growth solution S2. Over an 8-h period of continuous imaging, we initially observe surface roughening owing to the formation of 3-dimensional gel-like islands followed by a progressive smoothening of the surface due to the rastering of the AFM tip.

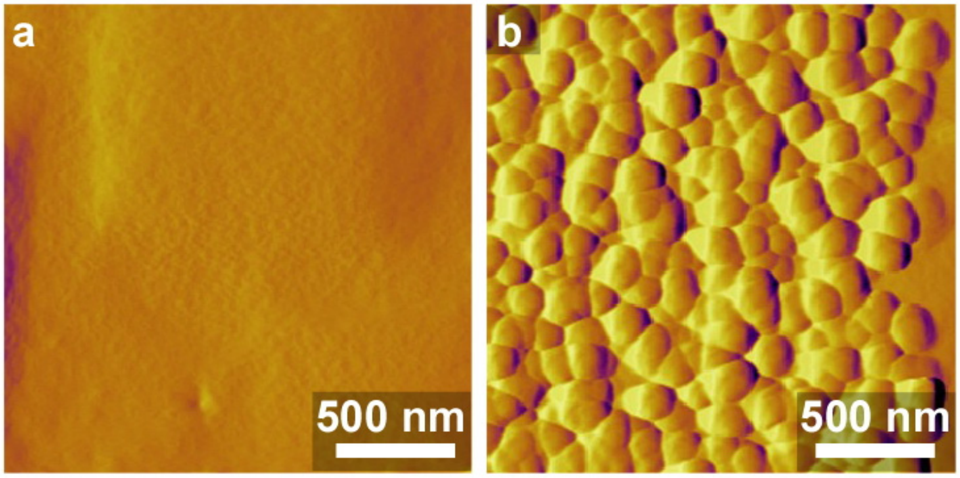

Supplementary Figure 7 │ Image depicting AFM tip effects. a. High resolution AFM amplitude mode image of the smooth area in figure 2d (region I) with its corresponding narrow height distribution in figure 2e. **b.** High resolution amplitude mode image of the rough area in figure 2d (region II) with its corresponding broad height distribution in figure 2e.

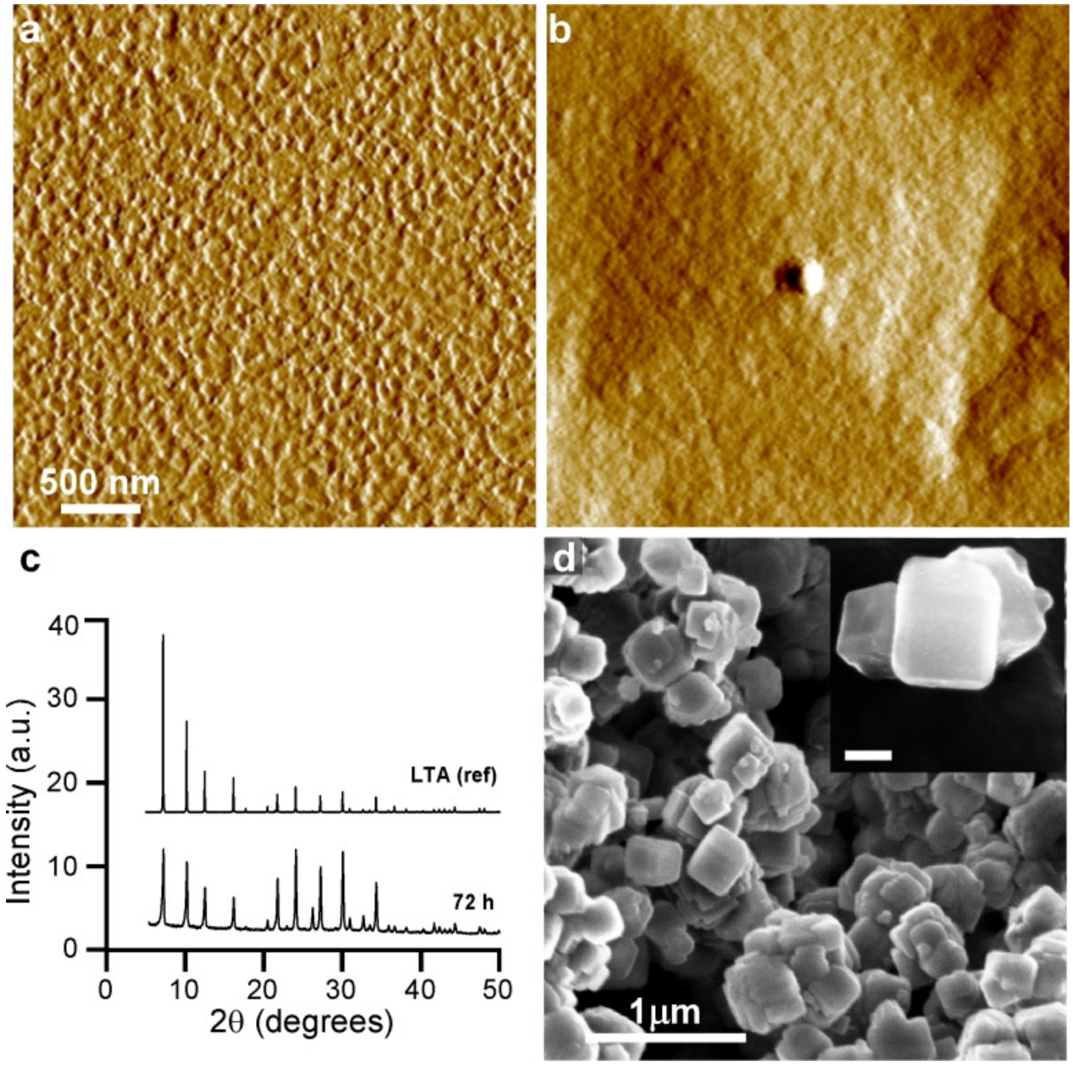

Supplementary Figure 8 | LTA crystallization at 35°C. a and b. AFM amplitude mode images of the LTA samples used for XPS analysis in Supplementary Table 2. Images are shown for *ex situ* crystallization in a S2 growth solution after (a) 2 h and (b) 12 h of heating. **c.** Powder X-ray diffraction pattern of the solid product extracted from a LTA growth mixture that was heated for 72 h. Comparison of peaks to the reference pattern of LTA (top pattern) reveal a fully crystalline product without any detectable impurity. **d.** Scanning electron micrograph of LTA crystals obtained from bulk crystallization. (inset) High magnification image of a representative crystal with cubic habit (scale bar is equal to 200 nm).

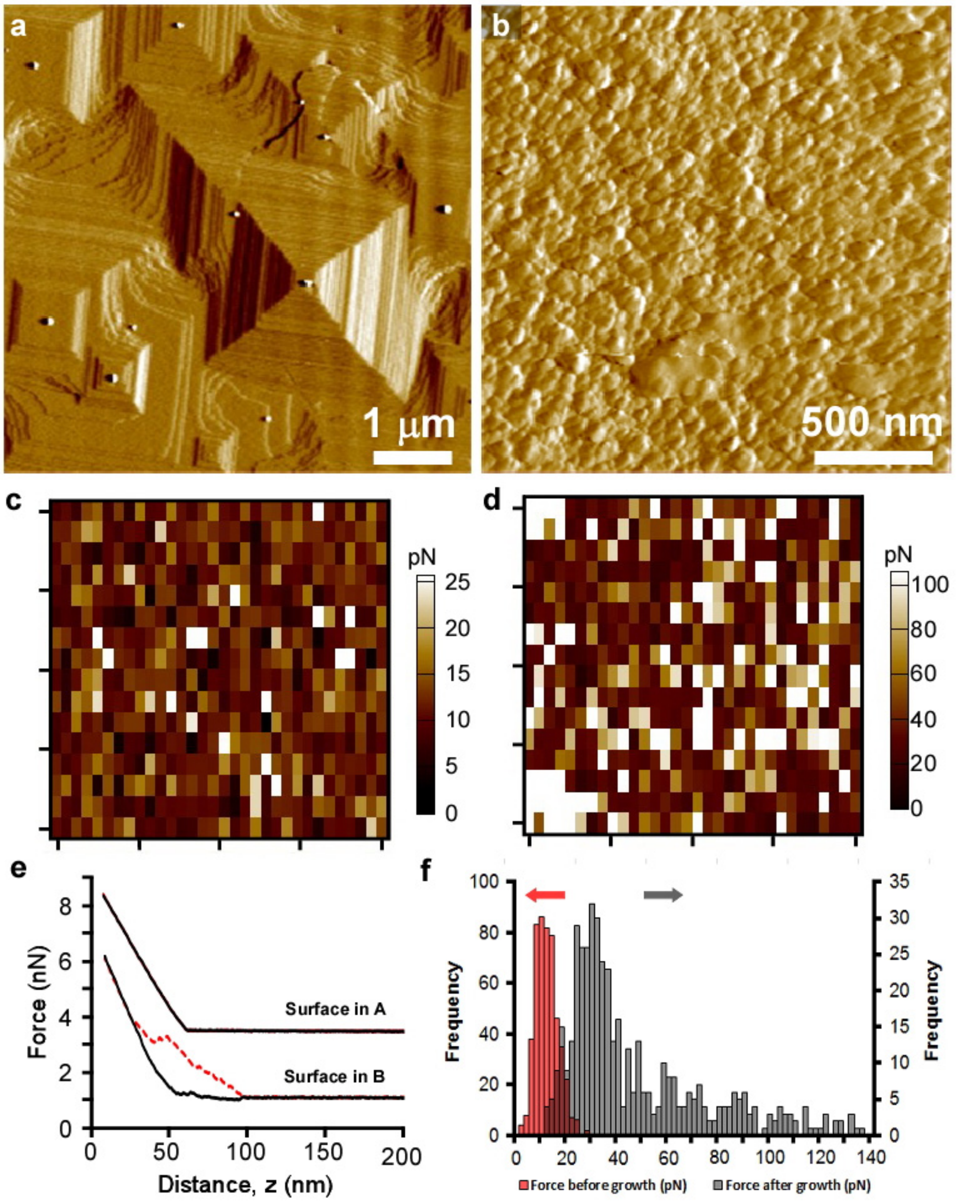

Supplementary Figure 9 │ Chemical force microscopy before and after growth. a and b. AFM deflection mode images of a LTA crystal surface (a) before and (b) after growth in solution S2, showing pristine and rough surfaces, respectively. **c and d.** Discretized force measurements (units of piconewtons) for sample areas corresponding to images a and b, respectively. The scan areas for CFM maps are 4 x 4 μm^2 and 2 x 2 μm^2 for pristine and rough surfaces, respectively. **e.** Representative approach-retract profiles from CFM analysis of surfaces in image a (top) and image b (bottom). The approach and retract curves are represented by dashed red lines and solid black lines, respectively. **f.** Histograms of the unbinding force measured between a Cr-Au coated silicon nitride AFM tip and LTA crystal surfaces before growth (red, left y-axis) and after growth (grey, right y-axis), corresponding to pristine and rough surfaces, respectively. The rough surface covered with gel-like islands exhibits larger (and a broader distribution of) tip-substrate unbinding force.

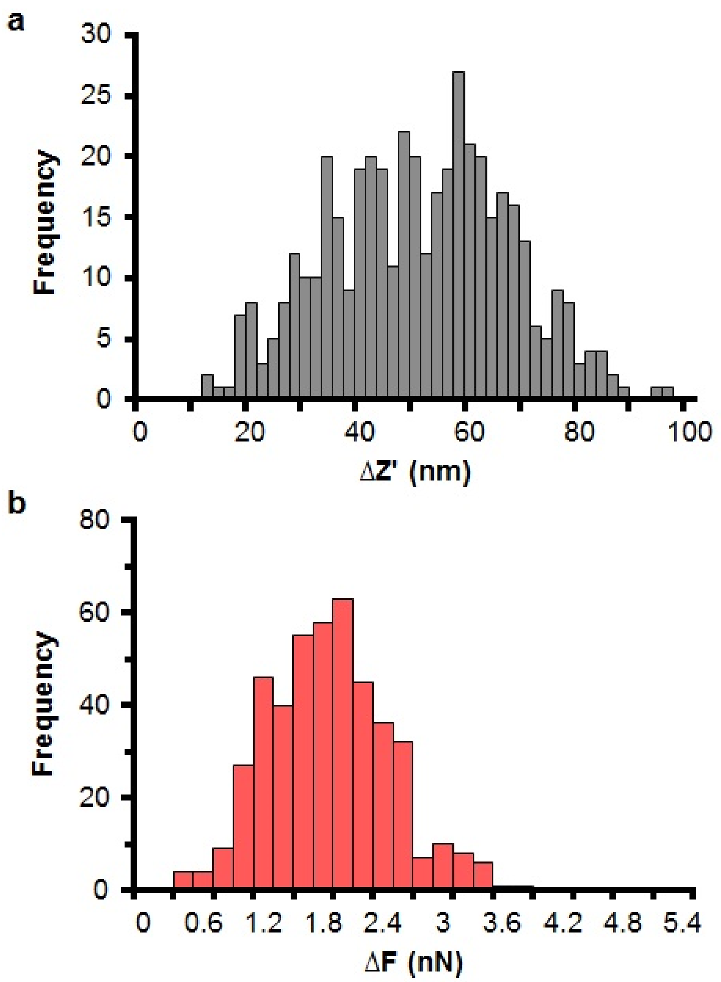

Supplementary Figure 10 | Hysteresis analysis between approach and retraction curve. a. Distribution of approach distances $\Delta z'$ for CFM measurements of rough LTA surfaces covered with gel-like islands. Measurements were made on samples heated in solution S2 at 35°C for 4 h, then cooled to room temperature prior to analysis. **b.** The corresponding distribution of breakpoint forces ΔF for the nonlinear approach curve. For definitions of $\Delta z'$ and ΔF, refer to figure 2f.

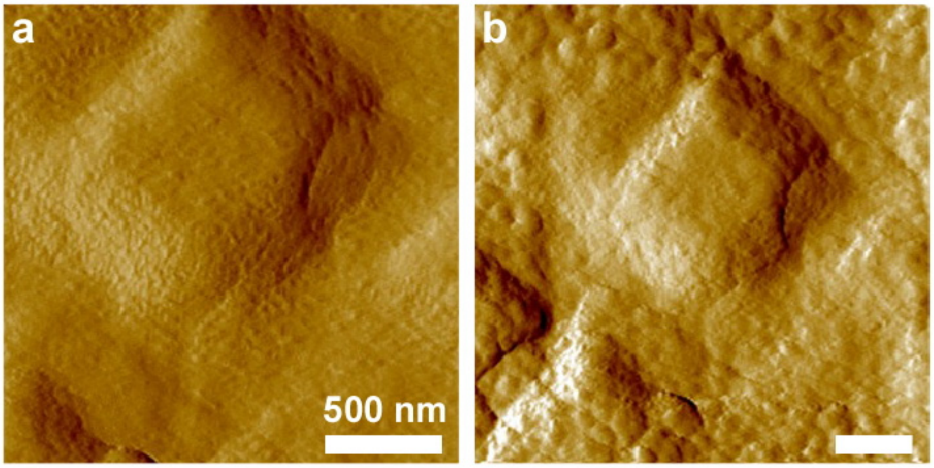

Supplementary Figure 11 | Tip effect at intermediate saturation and low temperature. a. Time-resolved AFM amplitude mode image showing an area after continuous scanning in growth solution S3 at 35°C. **b.** An enlarged scan area with the original smooth region in image a surrounded by rough areas.

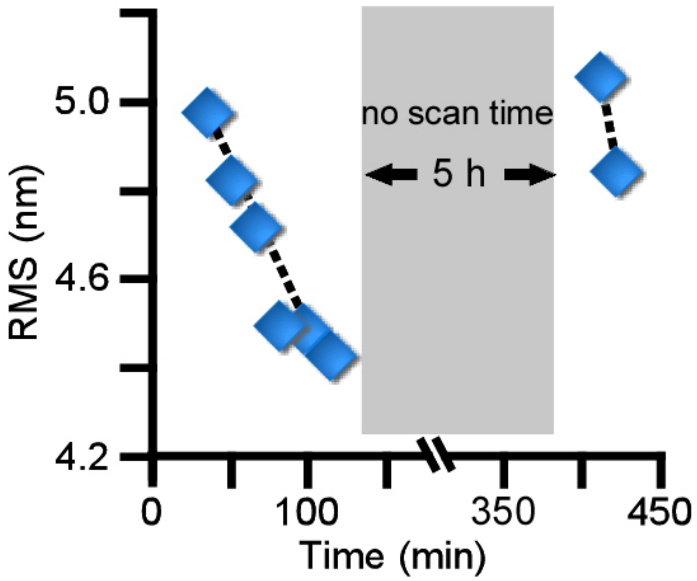

Supplementary Figure 12 | Evolution of surface roughness. Changes in the root mean squared (RMS) roughness of a LTA crystal surface as a function of time for a crystal substrate grown in solution S3. The substrate was heated at 35°C for 30 min prior to continuous AFM imaging. The RMS roughness decreases with continuous imaging owing to tip-induced smoothening of the surface. For a period of time (denoted by the grey shaded area), the AFM tip was removed from the scanning area, and then returned to the same area. Reimaging the same area resulted in a return to the original RMS, thus indicating a "recovery" to a roughened interface that once again begins to smoothen with continuous imaging. Dashed lines are interpolations to help guide the eye.

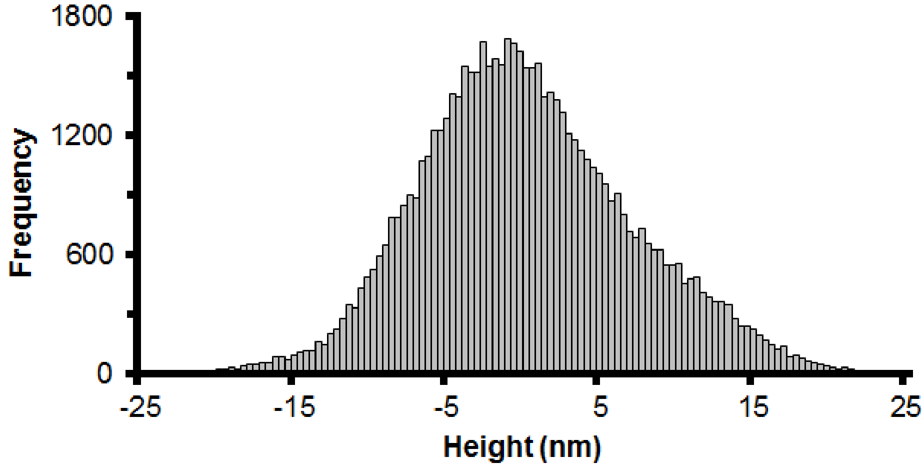

Supplementary Figure 13 | Height histogram of nanoparticle deposits. Height distribution of islands on the LTA crystal surface shown in figure 3a. The substrate was heated in growth solution S2 for 1 h at 45°C prior to analysis. The standard deviation of island height obtained using a Gaussian fit is ca. 6 nm (refer to Supplementary Note 1).

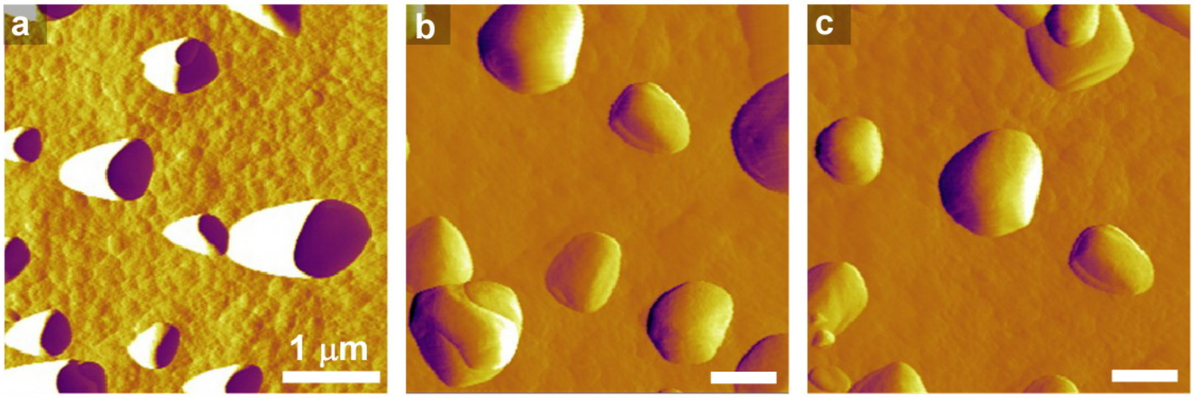

Supplementary Figure 14 | AFM images at high temperature and high supersaturation. a. AFM amplitude image of the same area shown in figure 3e. Poor tracking of large feature heights in tapping mode (i.e., light surface engagement) leads to the appearance of elongated islands (white features). **b and c.** Deflection images of similar areas taken in contact mode (i.e., harder surface engagement) show proper tracking of these features on the surface. Comparison of surfaces before and after continuous imaging show no visible signs of AFM tip effects that were observed at lower temperature. Note that all of the *in situ* images reported in the manuscript were taken in tapping mode to minimize the potential effects of the AFM tip.

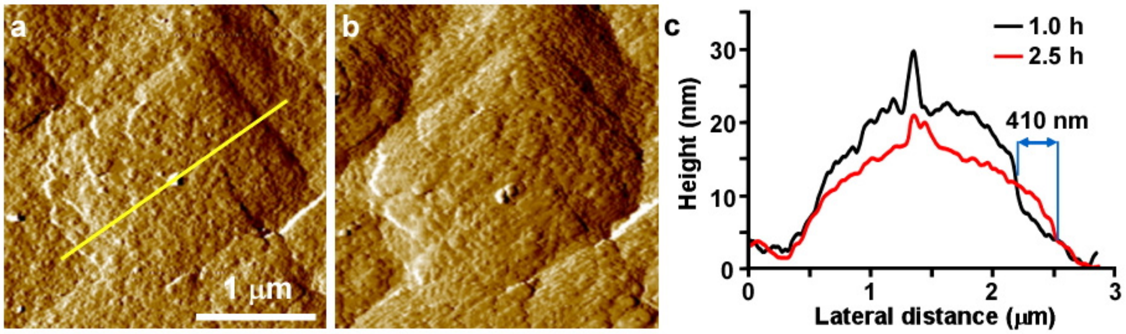

Supplementary Figure 15│ Growth of hillocks. a and b. AFM amplitude mode images of a crystal surface after (a) 1 h and (b) 2.5 h of imaging at 45°C in growth solution S4. There is a noticeable increase in the width of the hillock during this time. **c.** Height profiles along the yellow line in image a reveal an increase in the length of the hillock (Δx = 410 nm). Measurement of hillock height in the z-direction are challenging owing to the changing baseline (i.e., all surfaces within the imaging area are growing by the generation and spreading of layers).

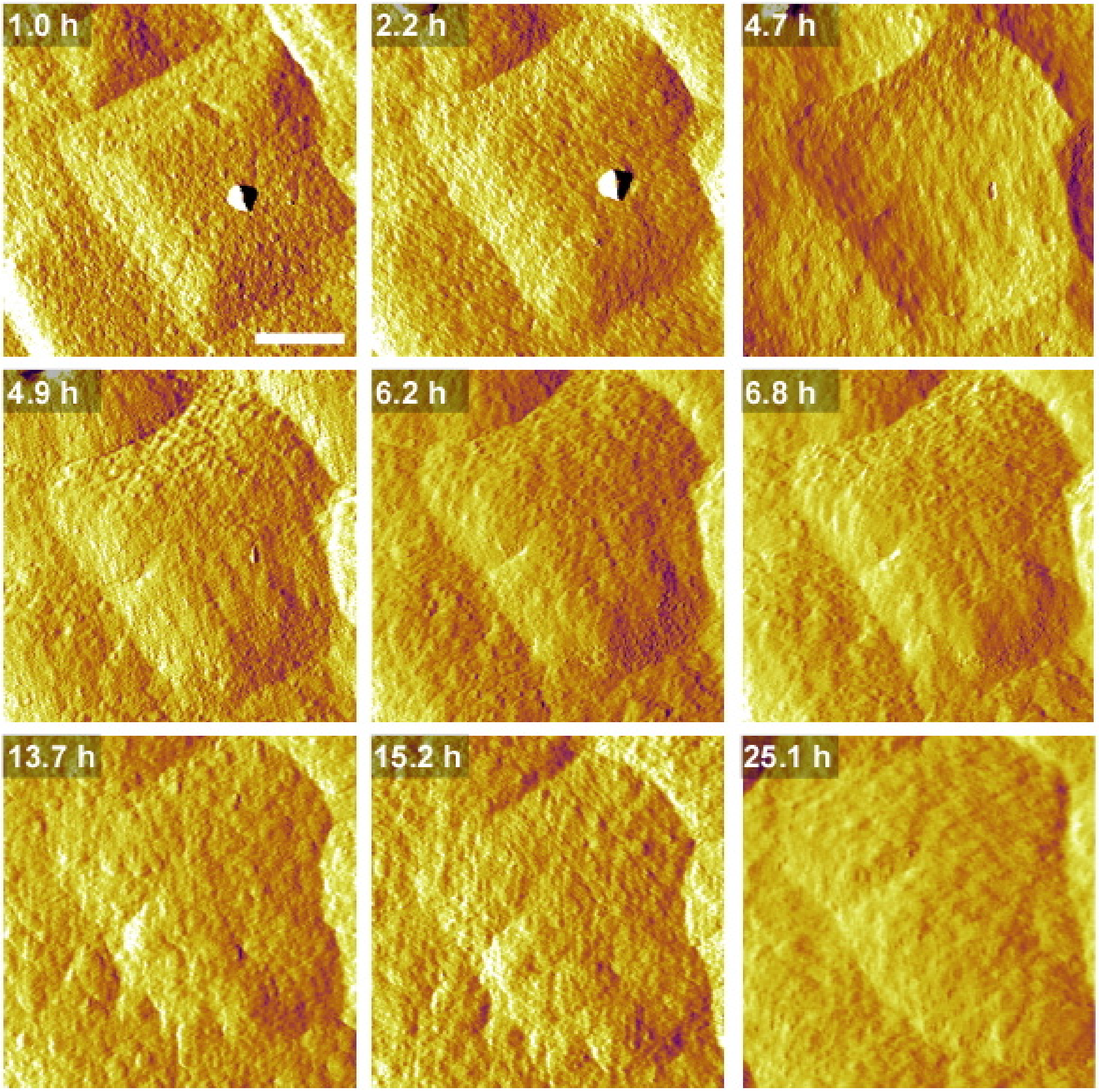

Supplementary Figure 16 | Evolution of a crystal surface at high temperature and moderate supersaturation. Time-resolved AFM amplitude mode images from Supplementary Movie 4 of a crystal surface grown in solution S3 at 45°C. After introducing the growth solution and increasing the temperature to the set point, we observe that the surface is covered with small islands. The surface smoothens with time, not as a result of the AFM tip, but due to the spreading of layers. Roughening occurs by the continual generation and spreading of new layers, which gives rise an interface that resembles kinetic roughening in classical theories. The scale bar is equal to 1 μm. The imaging time is listed on each micrograph.

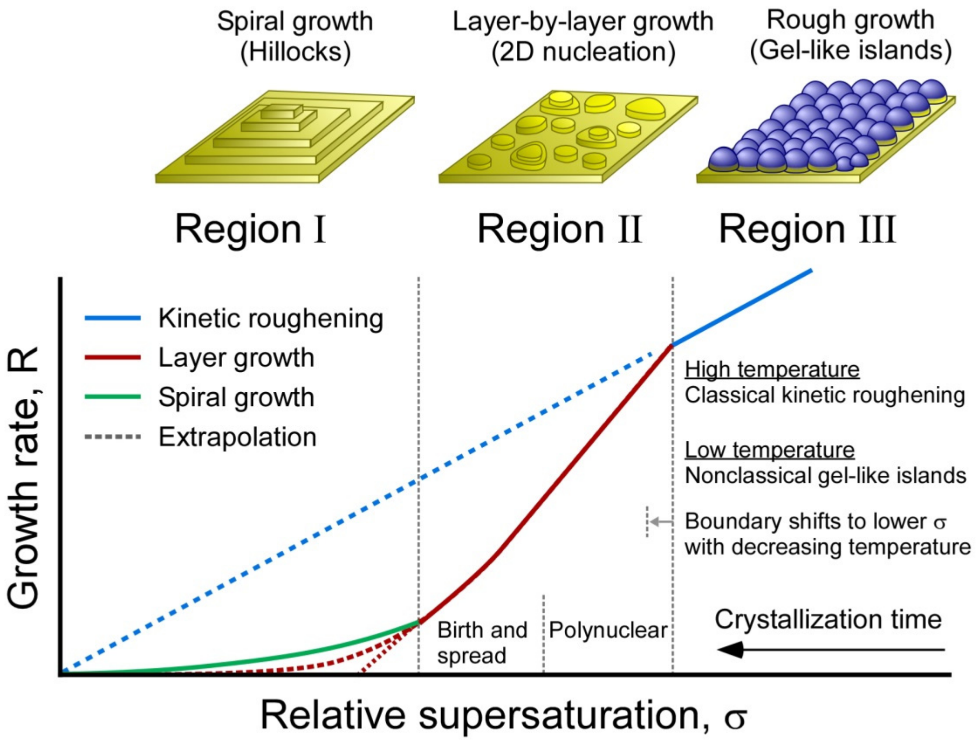

Supplementary Figure 17 │ Growth regimes as a function of supersaturation. Illustrative renderings of crystal growth mechanisms as a function of the relative supersaturation σ. (bottom) Growth rate dependency on σ (solid lines) for each regime according to the classical theories described in Supplementary Note 2. Crystal growth in region I (low σ) occurs by layers emanating from spiral dislocations, consistent with images of crystal surfaces removed from saturated growth solution (Fig. 1f). Crystal growth in region II (moderate σ) occurs by the formation and propagation of 2D layers (Fig. 4) via a birth and spread or polynuclear pathway. Growth in region III (high σ) occurs by kinetic roughening at high temperature, akin to classical models. At low temperature, growth occurs by a unique nonclassical mode of action wherein roughness derives from the formation of 3D gel-like islands (Fig. 2).

Supplementary Table 1. Elemental analysis of growth solutions using ICP-OES.

Solution	Concentration (M) [a], [b]			pH
	Si	Al	Na	
S2	0.025	0.292	2.81	13.7
S3	0.011	0.228	2.96	13.7
S4	0.009	0.218	2.90	13.7
S24 [c]	0.007	0.204	2.96	13.7

[a] Obtained by ICP-OES analysis (Intertek-Whitehouse)

[b] The water content changes by less than 0.1 wt%

[c] Approximate solubility of zeolite A; note that the supersaturation of zeolite growth solutions is difficult to define[31] owing to the presence of various oligomeric species and amorphous precursors

Supplementary Table 2. Elemental analysis (Si/Al ratio) of crystals before and during an intermediate stage of growth.

Sample / Growth Conditions	Si/Al Ratio [b]	
	EDX	XPS
LTA crystals	1.0	1.1
Ex situ growth (S2, 45 °C, 2 h) [a]	1.0	0.9
Ex situ growth (S2, 35 °C, 2 h) [a]	1.0	0.7
Ex situ growth (S2, 35 °C, 12 h) [a]	1.0	1.1

[a] LTA crystals (ca. 10 mg) were suspended in a S2 growth solutions (ca. 3 g) that was heated to the set point temperature. Samples were removed either after 2 h of heating (i.e., intermediate stage of growth) or after 12 h of heating (i.e., complete growth). The samples were imaged by AFM (Supplementary Fig. 8a and b) to confirm the presence of rough and layered surface features, respectively.

[b] Energy dispersive X-ray spectroscopy (EDX) data is an estimate of the bulk (overall) crystal composition while X-ray photoelectron spectroscopy (XPS) is a surface-sensitive technique that estimates the composition of exterior regions of the particle.

Supplementary References

1. De Yoreo, J. J. & Vekilov, P. G. in *Biomineralization* Vol. 54 *Reviews in Mineralogy & Geochemistry* (eds P. M. Dove, J. J. DeYoreo, & S. Weiner) 57-93 (Mineralogical Soc America, 2003).
2. Penn, R. L. & Banfield, J. F. Imperfect oriented attachment: Dislocation generation in defect-free nanocrystals. *Science* 281, 969-971 (1998).
3. Chung, J. *et al.* Molecular modifiers reveal a mechanism of pathological crystal growth inhibition. *Nature* 536, 446-450 (2016).
4. Teng, H. H., Dove, P. M. & De Yoreo, J. J. Kinetics of calcite growth: Surface processes and relationships to macroscopic rate laws. *Geochim. Cosmochim. Acta* 64, 2255-2266 (2000).
5. Rimer, J. D. *et al.* Crystal growth inhibitors for the prevention of L-Cystine kidney stones through molecular design. *Science* 330, 337-341 (2010).
6. Reviakine, I., Georgiou, D. K. & Vekilov, P. G. Capillarity effects on crystallization kinetics: Insulin. *J. Am. Chem. Soc.* 125, 11684-11693 (2003).
7. Vekilov, P. G. What determines the rate of growth of crystals from solution? *Cryst. Growth Des.* 7, 2796-2810 (2007).
8. Markov, I. V. Crystal Growth for Beginners. *World Scientific* 2nd Edition (1995).
9. Lovette, M. A. & Doherty, M. F. Predictive modeling of supersaturation-dependent crystal Shapes. *Cryst. Growth Des.* 12, 656-669 (2012).
10. Evans, J. W., Thiel, P. A. & Bartelt, M. C. Morphological evolution during epitaxial thin film growth: Formation of 2D islands and 3D mounds. *Surf. Sci. Rep.* 61, 1-128 (2006).
11. De Yoreo, J. J., Land, T. A. & Dair, B. Growth morphology of vicinal hillocks on the {101} face of KH_2PO_4: From step-flow to layer-by-layer growth. *Phys. Rev. Lett.* 73, 838-841 (1994).
12. Heringdorf, F., Reuter, M. C. & Tromp, R. M. Growth dynamics of pentacene thin films. *Nature* 412, 517-520 (2001).
13. Tersoff, J., Van der Gon, A. D. & Tromp, R. Critical island size for layer-by-layer growth. *Phys. Rev. Lett.* 72, 266 (1994).
14. Liu, X. Y., Vanhoof, P. & Bennema, P. Surface roughening of normal alkane crystals: solvent dependent critical behavior *Phys. Rev. Lett.* 71, 109-112 (1993).
15. Liu, X. Y. & Bennema, P. Foreign body induced kinetic roughening: Kinetics and observations. *J. Chem. Phys.* 115, 4268-4274 (2001).
16. Sleutel, M., Maes, D., Wyns, L. & Willaert, R. Kinetic roughening of glucose isomerase crystals. *Cryst. Growth Des.* 8, 4409-4414 (2008).
17. Gorti, S., Forsythe, E. L. & Pusey, M. L. Kinetic roughening and energetics of tetragonal lysozyme crystal growth. *Cryst. Growth Des.* 4, 691-699 (2004).
18. Gorti, S., Forsythe, E. L. & Pusey, M. L. Growth modes and energetics of (101) face lysozyme crystal growth. *Cryst. Growth Des.* 5, 473-482 (2005).
19. Jetten, L., Human, H. J., Bennema, P. & Vandereerden, J. P. On the observations of the roughening transition of organic crystals, growing from solution. *J. Cryst. Growth.* 68, 503-516 (1984).
20. Liu, X. Y. & Bennema, P. Observations on the roughening transition and the influence on morphology of crystals. *J. Cryst. Growth.* 139, 179-189 (1994).

21. Bennema, P. Morphology of crystals determined by alpha factors, roughening temperature, F faces and connected nets. *J. Phys. D-Appl. Phys.* 26, B1-B6 (1993).
22. Cuppen, H. M., Meekes, H., van Enckevort, W. J. P., Vlieg, E. & Knops, H. J. F. Nonequilibrium free energy and kinetic roughening of steps on the Kossel(001) surface. *Phys Rev B* 69 (2004).
23. Halpinhealy, T. & Zhang, Y. C. Kinetic roughening phenomena, stochastic growth directed polymers and all that. Aspects of multidisciplinary statistical mechanics. *Phys. Rep.-Rev. Sec. Phys. Lett.* 254, 215-415 (1995).
24. Cuppen, H. M., Meekes, H., van Enckevort, W. J. P. & Vlieg, E. Kink incorporation and step propagation in a non-Kossel model. *Surf Sci* 571, 41-62 (2004).
25. Majaniemi, S., AlaNissila, T. & Krug, J. Kinetic roughening of surfaces: Derivation, solution, and application of linear growth equations. *Phys Rev B* 53, 8071-8082 (1996).
26. Salditt, T. & Spohn, H. Kinetic roughening of a terrace ledge. *Phys. Rev. E* 47, 3524-3531 (1993).
27. Jeong, H. C. & Williams, E. D. Steps on surfaces: experiment and theory. *Surf. Sci. Rep.* 34, 171-294 (1999).
28. Spinner, M. A., Watkins, R. W. & Goldbeckwood, G. Simulation of growth and surface roughening of polymer single crystals. *J. Chem. Soc.-Faraday Trans.* 91, 2587-2592 (1995).
29. Burton, W. K., Cabrera, N. & Frank, F. C. *The growth of crystals and the equilibrium structure of their surfaces*. Vol. 243 (1951).
30. Elwenspoek, M. & Vandereerden, J. P. Kinetic roughening and step free energy in the solid-on-solid model and on naphthalene crystals. *J. Phys. A-Math. Gen.* 20, 669-678 (1987).
31. Cubillas, P. *et al.* AFM and HRSEM invesitigation of zeolite A crystal growth. Part 1: In the absence of organic additives. *J. Phys. Chem. C* 115, 12567-12574 (2011).
