# Supplementary material for: Transient modes of zeolite surface growth from 3D gel-like islands to 2D single layers
Source: Nat Commun. 2018 May 29;9:2129. doi: 10.1038/s41467-018-04296-4 (PMC5974312; doi:10.1038/s41467-018-04296-4)
Supplement: Supplementary file 2 — Description of Additional Supplementary Files [file 41467_2018_4296_MOESM2_ESM.pdf]

Description of Additional Supplementary Files

File Name: Supplementary Movie 1
Description: Intermediate supersaturation and low temperature. Time-resolved amplitude mode AFM images of a LTA crystal surface in S3 growth solution heated at 35°C over a period of 1.5 h. The interface is initially covered with gel-like islands; however, continuous scanning at low supersaturation and at low temperature reveals a progressive smoothening of the surface. The scanning area is 1.9 x 1.9 μm^2.

File Name: Supplementary Movie 2
Description: High supersaturation and high temperature. Time-resolved amplitude mode AFM images of a LTA crystal surface in S2 growth solution heated at 45°C over a period of 5 h. This video captures nanoparticle deposition on the crystal interface in a growth medium at high supersaturation and high temperature. Comparison of the scanned area (after continuous imaging) with the surrounding regions of the sample surface reveals negligible AFM tip effects. The scanning area is 3.5 x 3.5 μm^2.

File Name: Supplementary Movie 3
Description: High supersaturation and high temperature. Video compiled from time-elapsed deflection mode AFM images of a LTA crystal surface in S2 growth solution heated at 45°C (total imaging time = 5 h). The surface was initially imaged in tapping mode, but was switched to contact mode prior to collecting the video in order to improve the tracking of surface features of a single nanoparticle deposit. Time-resolved snapshots show nanoparticle faceting and growth by what appears to be molecule addition. The scanning area is 2.2 x 2.2 $\mu m^{2.}$

File Name: Supplementary Movie 4
Description: Low supersaturation and High temperature. Video compiled from time-elapsed amplitude mode AFM images of a LTA crystal surface in S4 growth solution heated at 45°C (total imaging time = 1.5 h). Growth at low supersaturation and high temperature shows the generation of 2-dimensional layers via a classical pathway. The scanning area is 3.4 x 3.4 μm^2..

File Name: Supplementary Movie 5
Description: Intermediate supersaturation and high temperature. Video showing sequential amplitude mode AFM images of a LTA crystal surface growing in S3 solution heated at 45°C for a total of 5 h. The generation of many nuclei leads to a roughened crystal surface, akin to classical theories postulated for kinetic roughening. The scanning area is 3.8 x 3.8 μm^2.

File Name: Supplementary Movie 6
Description: Intermediate supersaturation and high temperature. Time-resolved amplitude mode AFM images of LTA crystal growth in a S3 solution heated at 45°C for a long period (ca. 20 h). Continuous imaging reveals alterations between rough and smooth surface features owing to the generation and spreading of layers, respectively. The scanning area is 4 x 4 μm^2.
